# Validation of an in vitro muscle platform to evaluate myogenesis and calcium handling in control and dystrophic human myotubes

**DOI:** 10.1038/s41598-025-31522-z

**Published:** 2025-12-17

**Authors:** Laura Mosqueira-Martín, Carolina Prendes-García, Camila Vesga-Castro, Pablo Marco-Moreno, Ainhoa Irastorza, Ander Izeta, Iratxe Madarieta, Itxaso Martí-Carrera, Jacobo Paredes, Adolfo López de Munain, Ainara Vallejo-Illarramendi

**Affiliations:** 1 Group of Neuroscience, Departments of Pediatrics and Neuroscience, Faculty of Medicine and Nursing, UPV/EHU, San Sebastian, Spain; 2 Neuromuscular Diseases Group, Biogipuzkoa HRI, San Sebastian, Spain; 3 CIBERNED, CIBER, Instituto de Salud Carlos III, Madrid, Spain; 4 Tecnun School of Engineering, University of Navarra, San Sebastian, Spain; 5 Biomedical Engineering Center, University of Navarra, Pamplona, Spain; 6 Stem Cells and Aging Group, Biogipuzkoa HRI, San Sebastian, Spain; 7 Advanced Therapies Unit, Hospital Universitario Donostia, Osakidetza, San Sebastian, Spain; 8 Tecnalia, Basque Research and Technology Alliance (BRTA), San Sebastian, Spain; 9 Pediatrics Group, Biogipuzkoa HRI, San Sebastian, Spain; 10 Pediatrics Department, Hospital Universitario Donostia, Osakidetza, San Sebastian, Spain; 11 Neurology Department, Hospital Universitario Donostia, Osakidetza, San Sebastian, Spain; 12 Department of Medicine, Faculty of Medicine, University of Deusto, Bilbao, Spain

**Keywords:** Biological techniques, Biotechnology, Cell biology, Stem cells

## Abstract

Electrical impedance has emerged as a powerful tool for real-time, label-free, and non-invasive monitoring of cellular processes. Here, we employed an impedance-based assay to characterize the myogenic process of control and dystrophic human myoblasts. First, we conducted a comprehensive analysis of control myoblast differentiation, assessing the effects of initial seeding density and various extracellular matrix coatings. We also evaluated the influence of electrode presence and current application, both of which improved myoblast alignment. Immortalized myoblasts from Duchenne muscular dystrophy patients exhibited marked alterations in early differentiation and maturation, which were readily detected via impedance measurements. We further compared two differentiation protocols using one control and one dystrophic representative cell line. While both protocols supported the formation of mature myotubes, impedance profiles differed depending on the culture medium. Notably, we identified the protocol with superior impedance profile reproducibility over the culture lifespan. Finally, we successfully assessed calcium homeostasis in control and dystrophic myotubes differentiated on 96-well impedance plates. Our findings underscore the potential of impedance-based assays for monitoring myogenesis and identifying disease-associated phenotypes. Moreover, 96-well impedance plates represent a robust tool for high-throughput and high-content functional analysis in muscle disease modeling and therapeutic screening.

**Supplementary Information:**

The online version contains supplementary material available at 10.1038/s41598-025-31522-z.

## Introduction

Skeletal muscle accounts for approximately 40–50% of the total body mass in adults. Its functional unit, the myofiber, is a multinucleated contractile cell formed by fusion of multiple myoblasts through a process known as myogenesis. Myogenesis is a complex process that occurs during embryogenesis and in response to muscle injury in adults and is regulated by cell-cell and cell-matrix interactions^1^. The extracellular matrix (ECM), which constitutes around 5% of the skeletal muscle mass, plays a crucial role in the regulation of muscle cell gene expression, proliferation, adhesion, and differentiation^2^.

Muscular dystrophies are a group of genetically inherited disorders characterized by progressive muscle weakness and waste^3^. Among these, Duchenne muscular dystrophy (DMD) is the most common form in childhood, affecting approximately one in 3500 to 5000 live male births^4^. DMD is caused by out-of-frame mutations in the *DMD* gene on the X chromosome, leading to the absence of the dystrophin protein. This results in severe and progressive muscle degeneration, which ultimately leads to premature death due to respiratory failure or cardiac complications. Currently, there is no specific treatment available for patients^5^.

Mouse and human myoblasts have been widely used as *in vitro* models to study skeletal muscle pathophysiology. Significant progress has been made to address the low maturation, limited survival, and high inter-individual variability commonly observed in human myotubes. An area of focus has been the selection of an optimal source of myogenic cell. Although satellite cells and primary myoblasts offer high physiological relevance, their use is limited by factors such as tissue availability, low cell yield, and restricted proliferative capacity^6^. Human immortalized myoblasts present a valuable alternative, offering a balance between biological relevance and experimental scalability^7–11^. In addition, several strategies have been implemented to enhance myotube maturation and mimic physiological stimuli such as exercise. These include exogenous electrical stimulation^12^, co-culture with spinal cord explants or human motoneurons^13,14^, and trophic factors^15^. Additionally, engineered 2D platforms incorporating polymeric coatings or micro-patterned surfaces have improved myotube alignment and maturation^16,17^. More recently, 3D *in vitro* models have been developed to better replicate native muscle structure^18^. While these systems hold great promise, their implementation requires specialized handling, significant time investment, and high costs. Critically, the high variability in myotube formation and function remains a major limitation, undermining their reproducibility and utility in disease modeling.

Impedance-based cellular assays have emerged as powerful tools for assessing various cellular processes, including adhesion, migration, proliferation, differentiation, and cytotoxicity^19^. These assays are particularly attractive for disease modeling, as they enable non-invasive, real-time monitoring of cell viability as well as cell-substrate and cell-cell interactions. Cellular impedance is influenced by several parameters, such as cell morphology, membrane properties, intercellular junctions, and ECM composition. We previously demonstrated the utility of impedance-based analysis to detect altered myogenesis in myoblasts derived from Myotonic Dystrophy type 1^11^. In the present study, we expand upon this work by systematically characterizing how key culture variables –including seeding density, ECM composition, and differentiation protocols– influence myogenic progression in human myoblasts. Importantly, we also apply this approach to model DMD using patient-derived immortalized myoblasts, further validating the platform’s relevance across neuromuscular disease contexts. Finally, by integrating impedance monitoring with high-throughput (HTP) calcium (Ca^2+^) imaging, we establish a functional phenotyping system that enables multiparametric *in vitro* assessment of skeletal muscle physiology and disease.

## Results

### Impedance-based characterization of myogenesis in control human myoblasts: impact of seeding density

**Fig. 1:**
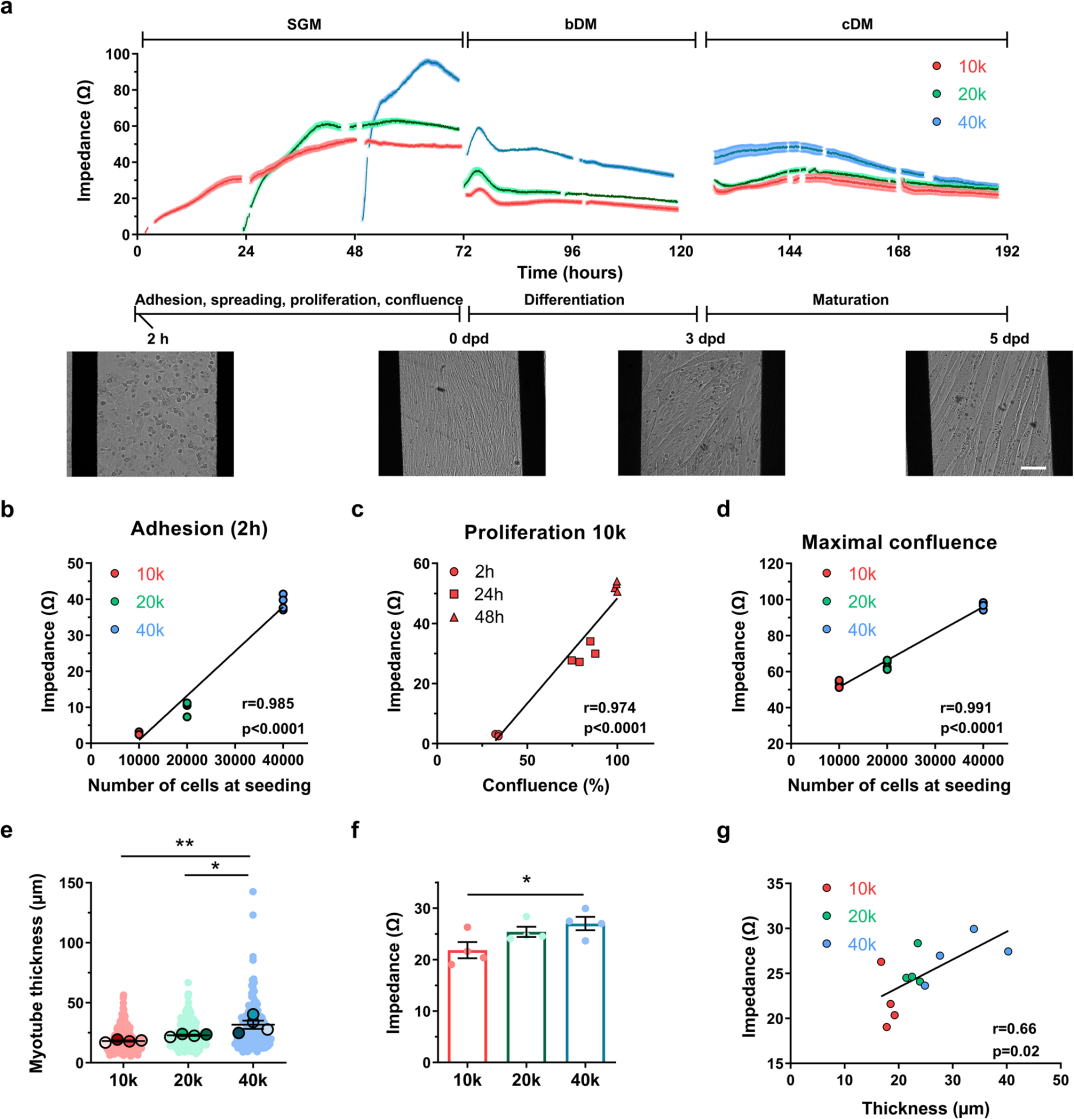
Impedance-based characterization of myogenesis in control human myoblasts: impact of seeding density. (**a**) Impedance profiles of Ctl1 myoblasts seeded at 10,000 (10 k), 20,000 (20 k) or 40,000 (40 k) cells per well. Insets display representative bright-field images captured at 2 h post-seeding, day post differentiation (dpd) zero, 3 dpd, and 5 dpd. Scale bar: 100 µm. SGM, Skeletal muscle cell Growth Medium; bDM, basic differentiation medium; cDM, complete differentiation medium. (**b**) Correlation between seeding density and impedance (Ω) at 2 h post-seeding. Pearson correlation coefficient (r) = 0.985, p < 0.0001. (**c**) Correlation between cell coverage (%) and impedance (Ω) at 10 k density across 2, 24, and 48 h. r = 0.974, p < 0.0001. (**d**) Correlation between seeding density and impedance (Ω) at confluence. r = 0.991, p < 0.0001. (**e**) Myotube thickness at 5 dpd: 18.08 ± 1.07 µm for 10 k, 22.86 ± 1.13 µm for 20 k, and 31.68 ± 6.88 µm for 40 k. Small dots represent individual myotubes; bold dots represent well averages. (**f**) Impedance (Ω) at 5 dpd. (**g**) Correlation between myotube thickness (µm) and impedance (Ω) at 5 dpd. r = 0.66, p = 0.02. Data in (**a**), (**e**), and (**f**) are expressed as mean ± SEM. n = 4 replicates per condition. *p < 0.05, **p < 0.01, One-way ANOVA post hoc Tukey’s multiple comparison test.

Myoblasts undergo several sequential steps *in vitro* before becoming mature myotubes: adhesion, spreading, proliferation, confluence, alignment, fusion and differentiation, and finally, maturation. Impedance measurements have previously been used to monitor this process using murine C2C12 cells. However, to our knowledge, this is the first study to systematically evaluate the use of impedance measurements as a tool to monitor the progression of myogenesis in human myoblasts. Therefore, we first aimed to validate the Impedance Module of the Maestro platform (Axion Biosystems) for myogenic profiling. Additionally, we sought to optimize cell seeding conditions to ensure a robust and reproducible impedance phenotype, which is essential for reliable interpretation and future disease phenotyping applications. To this end, we seeded the human immortalized control Ctl1 line on impedance plates (Z-plates) at varying densities of 10,000-40,000 cells per well (10k, 20k, 40k) and recorded the impedance profiles over the entire culture lifespan (Fig. 1a). The initial rise in impedance observed during the first hours corresponds to cell adhesion, spreading, and proliferation, with the peak corresponding to the point of maximal confluence. A subsequent drop in resistance likely reflects cellular reorganization prior to fusion and differentiation. A later increase in impedance is associated with myotube maturation. Finally, a decline in resistance at later time points may indicate myotube detachment and cell death.

We first examined the correlation between impedance measured 2 hours post-seeding and the initial cell number (Fig. 1b), finding a strong positive linear relationship, with a Pearson correlation coefficient (r) of 0.985 (p<0.0001). To further assess whether impedance reflects surface coverage, we analyzed cells seeded at 10k and correlated impedance 2-, 24-, and 48-hours post-seeding with the corresponding percentage of cell-covered area (Fig. 1c), obtaining a similarly strong positive correlation (r=0.974, p<0.0001). Representative bright-field images of Ctl1 myoblasts at these conditions are shown in Supplementary Fig. S1. With respect to confluence, we observed that maximum impedance values differed across the three seeding densities. These peak values also showed a linear correlation with cell number (Fig. 1d, r=0.991, p<0.001), suggesting that myoblasts seeded at 40k may reach a state of supra-confluence after 24h.

We next focused on characterizing myotubes at 5 days post differentiation (dpd). Analysis of myotube thickness (Fig. 1e) revealed that cultures seeded at 40k produced significantly thicker myotubes compared to those seeded at 10k (p<0.01) and 20k (p<0.05), with 44% and 29% reductions in thickness, respectively. Supplementary Fig. S2a shows representative bright-field images of these myotubes. This trend was also observed with impedance measurements, with 25% higher values in the 40k condition compared to the 10k condition (Fig. 1f, p<0.05). Consistent with these observations, we found a significant correlation between impedance and myotube thickness (Fig. 1g, r=0.66, p=0.02). This finding supports the use of impedance measurements as an indirect indicator of myotube thickness in myotube cultures in uncoated plates under varying seeding densities. Moreover, among the tested conditions, the 40k seeding density consistently generated the most mature myotube phenotype, both morphologically and in terms of impedance signal, supporting its use as the optimal condition for robust impedance-based myogenesis assessment.

### Impedance-based evaluation of the myogenic potential of porcine-derived decellularized matrices

The ECM plays a critical role in maintaining muscle homeostasis. In this study, we used impedance measurements to evaluate the myogenic potential of various ECM coatings, comparing them to uncoated plates (negative control). Specifically, we assessed the effects of porcine-derived decellularized adipose tissue (FATRIX) and skeletal muscle (MATRIX). As positive controls, we included commercially available gelatin and ECM-Gel.

**Fig. 2:**
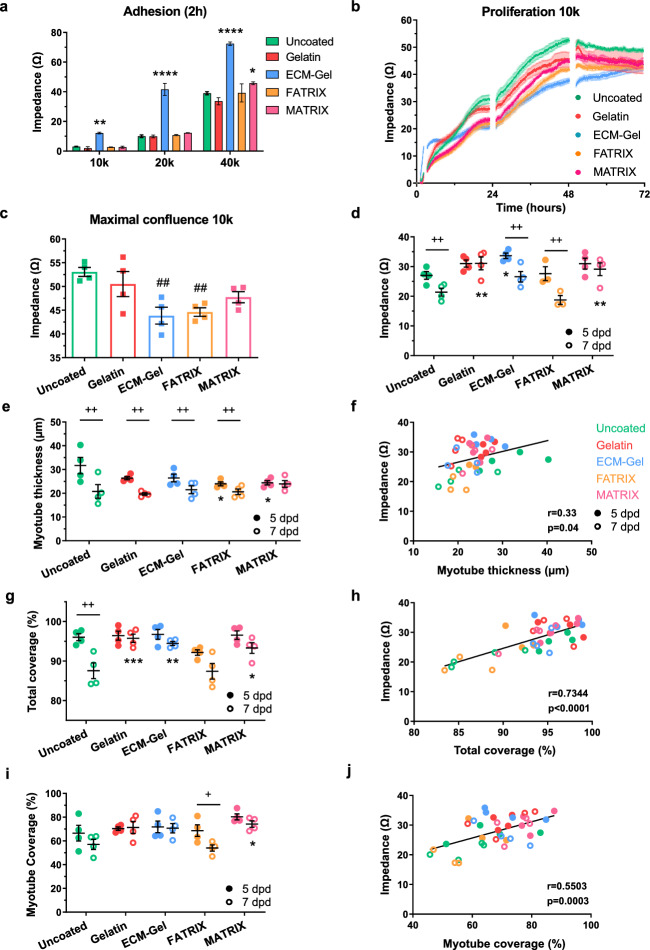
Impedance-based evaluation of the myogenic potential of different extracellular matrix coatings. (**a**) Impedance values (Ω) 2 h post-seeding of Ctl1 myoblasts seeded at 10,000 (10 k), 20,000 (20 k) or 40,000 (40 k) cells per well in uncoated plates or plates coated with Gelatin, ECM-Gel, or porcine-derived adipose tissue (FATRIX) or skeletal muscle (MATRIX). (**b**) Proliferation of cells seeded at 10 k measured by impedance (Ω). (**c**) Maximum impedance values (Ω) at confluence of cells seeded at 10 k. (**d**–**e**) Comparison of the Impedance values (**d**) and Myotube thickness (**e**) at 5 days post differentiation (dpd) and 7 dpd of cells seeded at 40 k. (**f**) Correlation between impedance (Ω) and myotube thickness at 5 dpd and 7 dpd. r, Pearson correlation coefficient. (**g**–**h**) Comparison of Total coverage (**g**) and Correlation between impedance (Ω) and total coverage (**h**) at 5 dpd and 7dpd. (**i**–**j**) Comparison of Myotube coverage (**i**) and Correlation between impedance (Ω) and myotube coverage (**j**) at 5 dpd and 7dpd. All data expressed as mean ± SEM. n = 4 replicates per condition. *p < 0.05, **p < 0.01, ***p < 0.001, ****p < 0.0001, Two-way ANOVA post hoc Dunnett’s multiple comparison test vs uncoated. ##p < 0.01, One-way ANOVA post hoc Dunnett’s multiple comparison test vs uncoated. + p < 0.05, +  + p < 0.01, Paired t test.

In terms of adhesion (2h), ECM-Gel significantly enhanced cell attachment compared to uncoated wells in 10k, 20k and 40k cell seeding densities (Fig. 2a, p<0.01 and 0.0001), with a 4-fold increase observed at the lower densities. MATRIX-coated wells also led to enhanced impedance levels when cells were seeded at 40k (p<0.05). In contrast, the highest proliferation was observed in uncoated wells (Fig. 2b). Regarding the time required to reach confluence, cells in uncoated wells reached this point fastest (48h), followed closely by those on gelatin and MATRIX (51h). Cells seeded onto FATRIX (66h), and ECM-Gel (71h) required a longer time to reach confluence. At the point of maximal confluence, uncoated wells exhibited the highest impedance values (Fig. 2c), while ECM-Gel showed the lowest (p<0.01), with 17% reduction compared to uncoated wells. Similarly, FATRIX-coated wells displayed lower impedance values (16%, p<0.01), whereas gelatin and MATRIX coatings did not differ from the uncoated condition.

We next evaluated impedance values of cells seeded at 40k cells/well at 5 dpd and 7 dpd (Fig. 2d). Gelatin and MATRIX were the only conditions without significant differences between time points. While at 5 dpd, ECM-Gel exhibited higher impedance values (25%) than uncoated wells (p<0.05), by day 7, significant differences were observed for both gelatin (45%) and MATRIX (36%) compared to uncoated wells (p<0.01). Representative bright-field images of the formed myotubes are shown in Supplementary Fig. S2b and c.

Regarding myotube thickness (Fig. 2e), we found that myotubes formed on uncoated wells at 5 dpd were significantly thicker compared to FATRIX (p<0.05) and MATRIX (p<0.05). We then compared myotube thickness at 5 dpd and 7 dpd and observed a significant reduction over time in all conditions except MATRIX, which remained unchanged. To gain further insights into myotube morphology, we analyzed the distribution of myotube diameters using a cumulative index approach (Supplementary Fig S3a), which revealed a significant shift toward thinner myotubes from 5 to 7 dpd in all conditions except MATRIX. This reduction was most pronounced in Uncoated and Gelatin-coated conditions (p<0.0001), followed by ECM-Gel (p<0.001) and FATRIX (p<0.01) coatings, whereas no significant change was detected in MATRIX coating. As for the correlation between myotube thickness and impedance (Fig. 2f), we found a significant but moderate correlation at 5 and 7 dpd (r=0.33, p=0.04). When analyzing each coating condition separately (Supplementary Fig S3b), only the uncoated wells showed a significant positive correlation between impedance and myotube thickness (r=0.8431, p=0.0086), consistent with previous observations. No significant correlations were found for the other coatings, although FATRIX exhibited a similar positive trend (r=0.6252). These results suggest that in uncoated wells, impedance changes closely reflect myotube morphological development, whereas in coated conditions this relationship becomes weaker, possibly due to the influence of the substrate on myoblast/myotube ratios.

In terms of total surface coverage (Fig. 2g), significant differences were observed between time points only in uncoated wells. At 7 dpd, coverage was significantly higher in gelatin (9%, p<0.001), ECM-Gel (8%, p<0.01), and MATRIX (7%, p<0.05) coatings compared to uncoated wells. In this case, we found a strong positive correlation between impedance and total coverage across coating conditions at 5 and 7 dpd (Fig. 2h, r=0.7344, p<0.0001). We further evaluated whether impedance also reflects myotube coverage. Myotube coverage was significantly reduced from 5 to 7 dpd in FATRIX (p<0.05) and was significantly higher at 7dpd in MATRIX compared to uncoated plates (Fig. 2i, p<0.05). Importantly, a significant positive correlation was observed between myotube coverage and impedance (Fig. 2j, r=0.5503, p=0.0003). These findings further support the use of impedance as a reliable indirect readout of total and myotube coverage under different coating conditions.

Considering the pronounced drop in myotube thickness in uncoated wells (34%), we further investigated if it was caused by cell detachment and loss of viability. Using a Live/Dead assay combined with Calcein staining, we found a moderate reduction in coverage (20%, non significant) that was accompanied by a great loss in total cell numbers (50%, p<0.01) at 7 dpd compared to 5 dpd. However, there was a moderate increase in the percentage of dead nuclei (20%, non significant). These results suggest that the impedance reduction from 5 to 7dpd is mainly due to myotube detachment, preferentially from thicker myotubes.

### Impact of plate substrate and impedance-related current application on myotube alignment and fusion

We next aimed to assess the impact of plate substrate and electrical current on the differentiation of Ctl1 myoblasts. To this end, we seeded 40k cells/well in a standard 96-well plate and in two Z-plates: one docked to the Maestro Edge and subjected to electrical current (Z-plate current), and another kept in a standard incubator, with no current (Z-plate no current).

**Fig. 3:**
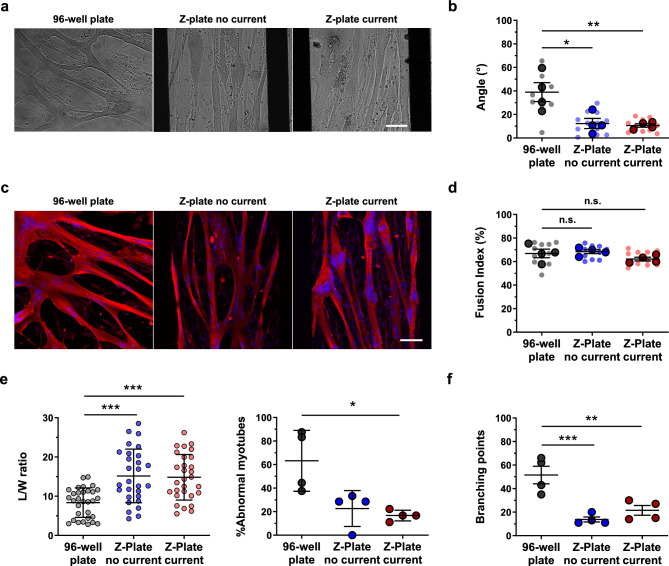
Impact of plate substrate and impedance-related current application on myotube alignment and fusion. (**a**) Representative bright-field images of Ctl1 cells at 5 days post differentiation (5 dpd) seeded at 40,000 cells per well in a standard 96-well plate, Z-plate without current, and Z-plate with current. Scale bar: 100 µm. (**b**) Myotube alignment (angle º) at 5 dpd. *p < 0.05, **p < 0.01, Ordinary One-way Anova Dunnett’s post hot multiple comparison test vs 96-well plate. (**c**) Representative immunofluorescence images of myotubes at 5 dpd in standard and Z-plates. Myotubes were stained with sarcomeric *α*-actinin (red) and DAPI (blue). Scale bar: 100 µm. (**d**) Fusion index (%) at 5 dpd. n.s., non significant, Ordinary One-way Anova Dunnett’s post hot multiple comparison test vs 96-well plate. (**e**) Left: length-to-width ratio (L/W ratio) of individual myotubes at 5 dpd. Right: Percentage of abnormal myotubes per well, defined as those with L/W ratios below 10. *p < 0.05, ***p < 0.001, Kruskal–Wallis Dunn’s post hot multiple comparison test vs 96-well plate. (**f**) Number of branching points per myotube at 5 dpd. **p < 0.01, ***p < 0.001, Ordinary One-way Anova Dunnett’s post hot multiple comparison test vs 96-well plate. Data in (**b**), (**d**), and (**f**) expressed as mean ± SEM. Data in (**e**) expressed as mean ± SD. n = 4 replicates per condition. Small dots in (**b**) and (**d**) represent individual fields of view; bold dots represent well averages.

Representative bright-field images of myotubes at 5 dpd in each condition are shown in Fig. 3a. Alignment analysis at this time-point revealed that myotube orientation was improved in both Z-plates compared to the standard plate (Fig. 3b; 39 ± 16 for 96-well plate, 12.3 ± 8.6 and p<0.05 for Z-plate no current, and 10.6 ± 2.9 and p<0.01 for Z-plate current), with an approximate 70% improvement in myotube alignment. We also evaluated the fusion index (FI), a widely used metric for assessing culture maturation. At 5 dpd, myotubes were stained for *α*-actinin and DAPI (Fig. 3c), and the percentage of nuclei within myotubes was calculated for each condition. While no significant differences in FI were observed between the Z-plates and the standard plate (Fig. 3d), the variability, as measured by the coefficient of variation (CV), was markedly higher in the standard plate (10.78%) compared to Z-plates, both with no current (4.89%) or with current applied (5.09%).

To further characterize myotube morphology, we analyzed the length-to-width ratio (L/W ratio), a parameter indicative of elongation and structural integrity. Z-plates, both with and without current, exhibited significantly higher L/W ratios compared to the standard plate (Fig. 3e, p<0.001), suggesting a more elongated and potentially more mature morphology. To assess the proportion of myotubes with abnormal morphology, we defined a threshold based on the distribution of L/W ratios observed in the Z-plates. Specifically, myotubes with a ratio below the 25th percentile of the Z-plates data (L/W ratio <10) were classified as morphologically abnormal. This data-driven approach allowed us to identify structures that deviate from the typical elongated phenotype, often appearing shorter and wider, which may reflect suboptimal differentiation. Interestingly, the Z-plate with current application showed a significantly lower percentage (p<0.05) of morphologically abnormal myotubes (73%) compared to the standard plate, further supporting the potential of impedance-based culture systems to promote more consistent and structurally refined myotube formation. Additionally, we quantified the number of branching points per myotube (Fig. 3f), a feature associated with irregular growth. Both Z-plates, regardless of current application, exhibited a significant reduction (p<0.01 for Z-plate current, and p<0.001 for Z-plate no current) in branching points compared to the standard plate, reinforcing the notion that impedance-based systems contribute to improved morphological organization.

### Impedance-based characterization of myogenesis in control and dystrophic human myoblasts

Next, we sought to determine whether this approach could also be applied to *in vitro* models of DMD. To this end, we employed several control and dystrophic immortalized myoblast lines (Supplementary Table S1). First, we confirmed the absence of dystrophin protein in dystrophic myotubes using capillary western blotting (Supplementary Fig. S4).

Before evaluating the myogenic process of dystrophic myoblasts using impedance, we first assessed whether these cells exhibited any impairments in fusion and/or maturation. To analyze fusion, myotubes at 5 dpd were stained for myosin heavy chain (MyHC) and DAPI. Quantification of the FI revealed no significant differences among the cell lines (Supplementary Fig. S5a). In parallel, maturation was evaluated by measuring MyHC protein levels. No significant differences in MyHC expression were detected across the different cell lines (Supplementary Fig. S5b–c), although there was a slight decrease in DMD lines compared to controls.

**Fig. 4:**
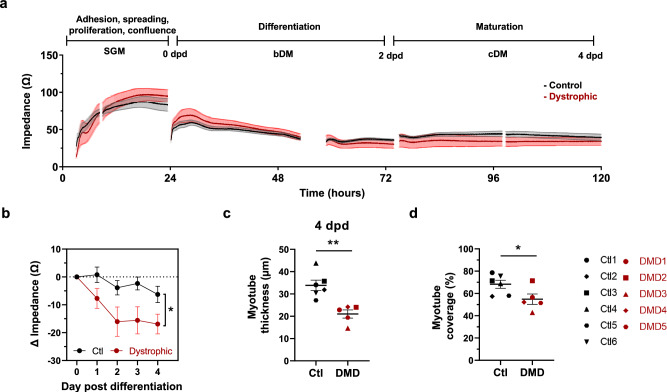
Impedance-based characterization of myogenesis in control and dystrophic human myoblasts. (**a**) Average impedance profiles of control (Ctl, n = 6 lines) and dystrophic (n = 5 DMD) myoblasts seeded at 40,000 cells per well in uncoated Z-plates in 6 replicates. (**b**) Change in impedance values (Ω) from 0 to 4 days post differentiation (dpd). Data are from n = 6 Ctl and n = 5 dystrophic cell lines from 3 independent experiments performed in 6 replicates. *p < 0.05, Two-way ANOVA, main effect of genotype. (**c**) Myotube thickness (µm) at 4 dpd. 5 replicates per cell line were analyzed. **p < 0.01 Unpaired T test. (**d**) Myotubes coverage (%) at 4 dpd. 1 replicate per cell line was analyzed. *p < 0.05, Unpaired T test. All data expressed as mean ± SEM. SGM, Skeletal muscle cell Growth Medium; bDM, basic differentiation medium; cDM, complete differentiation medium; dpd, days post differentiation.

Our next goal was to assess the myogenic process of these cells using impedance measurements, conducting three independent experiments in which cells were seeded at 40k cells/well in uncoated Z-plates. Under these conditions, the cultures became overconfluent after 24 hours. We deliberately chose uncoated plates to avoid masking the dystrophic phenotype, as coatings containing ECM components could potentially interfere with phenotypic expression. Moreover, our previous results demonstrated that uncoated impedance plates provide suitable support for the generation of mature myotubes. A representative impedance curve showing the differentiation of control and dystrophic myoblasts is presented in Fig. 4a.

We initially analyzed raw impedance values and found no significant differences between control and dystrophic lines regarding maximal confluence, or the time required to reach confluence, although dystrophic cells tended to show slightly higher values (Supplementary Fig. S6a). Similarly, no differences were observed in the impedance profiles during the differentiation phase (Supplementary Fig. S6b). However, analysis of impedance change (relative to 0 dpd) revealed that dystrophic cells exhibited significantly greater impedance loss during differentiation compared to control cells (Fig. 4b, p<0.05). Furthermore, evaluation of myotube thickness at 4 dpd revealed that dystrophic myotubes were significantly thinner (40%) than those in control cultures (Fig. 4c and Supplementary Fig. S6c, p<0.01). This reduction was accompanied by a significant decrease in myotube coverage (Fig. 4d, p<0.05).

**Fig. 5:**
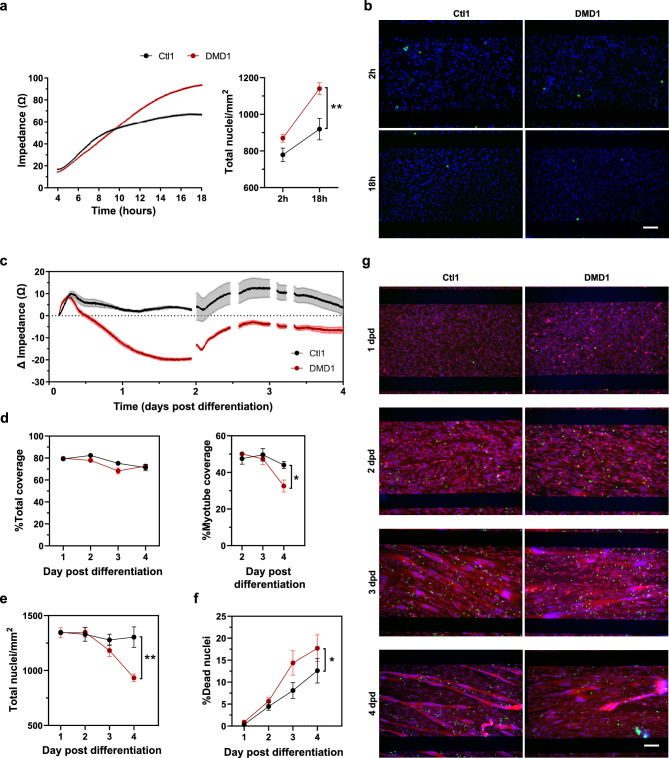
Pathological features underlying impedance differences between control and dystrophic cultures. (**a**) Impedance values (Ω) measured over 18 h after seeding (left), and quantification of cell number (Total nuclei/mm^2^) 2 and 18 h post-seeding (right) in Ctl1 (black) and DMD1 (red) myoblasts. (**b**) Representative fluorescence images showing all nuclei (blue) and dead nuclei (green) 2 and 18 h post-seeding. Scale bar: 100 µm. (**c**) Impedance change during differentiation from day 0 to day 4 post differentiation (dpd). (d) Total coverage (left) and myotube coverage (right) during differentiation. (**e**) Total nuclei count per mm^2^ from day 1 to 4 dpd. (**f**) Percentage of dead nuclei in myotubes. (**g**) Representative fluorescence images of Ctl1 and DMD1 cultures during differentiation, showing myotubes stained with Calcein (red), all nuclei (blue), and dead nuclei (green). Scale bar: 100 µm. All data are expressed as mean ± SEM. n = 5 replicates per condition. *p < 0.05, **p < 0.01 Two-way ANOVA, main effect of genotype.

To further investigate the mechanisms underlying the impedance differences between control and dystrophic cultures, we next examined additional pathological features in representative lines from each genotype (Ctl1 and DMD1). First, we evaluated whether the trend towards higher impedance values in dystrophic lines was related to higher proliferation. Impedance values measured 18 hours after seeding were higher in DMD1 myoblasts compared to Ctl1, correlating with the significantly higher cell number (%24) observed at this time point (Fig. 5a-b, p<0.01). This finding indicates that increased impedance of dystrophic lines at confluence (0 dpd) are likely due to higher proliferation rates compared to control cell lines. Regarding differentiation, DMD1 cells exhibited higher impedance drops from the first day of differentiation (Fig. 5c). Interestingly, this early impedance drop observed in dystrophic myotubes does not seem to be related to a loss of cell coverage or cell numbers, as observed in Fig. 5d, e. Indeed, total surface coverage remained comparable between genotypes from 1 to 4 dpd. However, a significant reduction in myotube coverage (26%) was observed in the DMD1 line at 4 dpd (Fig. 5d, p<0.05), consistent with the marked decrease in total nuclei number (Fig. 5e, p<0.01). Furthermore, Live/Dead staining revealed a significantly higher proportion of dead nuclei in DMD1 myotubes compared to Ctl1, beginning at 3dpd (Fig. 5f, p<0.05). Altogether, these results suggest that the early impedance alterations observed at days 1–2 dpd are primarily associated with morphological differences that do not impact total cell number, whereas from day 3 onward, increased cell death and subsequent myotube detachment contribute to the observed decline in impedance. Representative images of Ctl1 and DMD1 cells during the differentiation phase are shown in Fig. 5g.

### Optimization of culture protocols: comparative and reproducibility analyses

Although our standard differentiation protocol revealed a clear dystrophic phenotype using impedance-based measurements, it involves the sequential use of three distinct media with markedly different compositions. This complexity may introduce variability that can hinder the direct comparison of impedance profiles across experiments or conditions. In contrast, differentiation protocols commonly described in the literature often rely on a single basal medium used throughout both proliferation and differentiation stages –initially supplemented with serum to promote cell growth and later modified by serum withdrawal and the addition of pro-differentiation factors. Given these differences, our next objective was to compare our in-house protocol (Protocol I)^20,21^ with a simplified, literature-based alternative (Protocol II)^22^, using one representative myoblast line per genotype. This comparison aimed to determine how the choice of differentiation strategy influences impedance readouts and assay reproducibility.

**Fig. 6:**
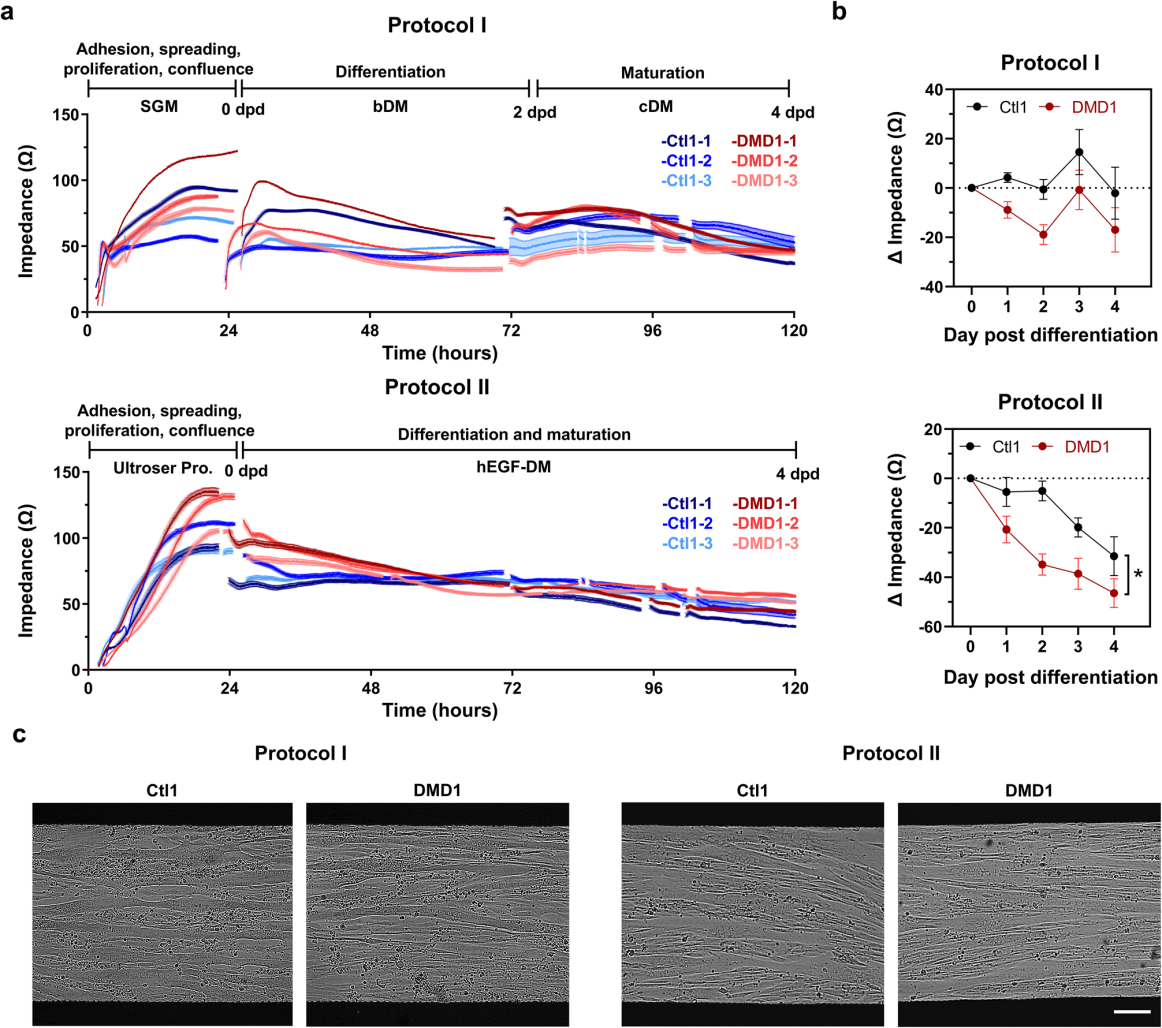
Impact of culture protocol on impedance values. (**a**) Impedance curves of control (Ctl1, blue) and Duchenne (DMD1, red) myoblasts seeded at 40,000 cells per well in uncoated impedance plates, cultured using either Protocol I (top) or Protocol II (bottom) across three independent experiments (− 1, − 2, − 3). Protocol I: SGM (Skeletal muscle cell Growth Medium), bDM (basic differentiation medium), and cDM (complete differentiation medium). Protocol II: Ultroser proliferation (Pro.) and hEGF-DM (human epidermal growth factor differentiation medium). dpd, days post differentiation. (**b**) Change in impedance values (Ω) from 0 to 4 dpd using Protocol I (top) or Protocol II (bottom). (**c**) Representative bright-field images of Ctl1 and DMD1 myotubes at 4 dpd differentiated in Protocol I (left) or Protocol II (right). Scale bar: 100 µm. All data expressed as mean ± SEM. Data are from n = 2 cell lines in 5–15 replicates and repeated 3 times. *p < 0.05, Two-way ANOVA, main effect of genotype.

To this end, Ctl1 and DMD1 cells were seeded in uncoated Z-plates at 40k cells/well and cultured using either Protocol I or Protocol II. Three independent experiments were conducted to assess reproducibility. The impedance profiles for Ctl1 (blue) and DMD1 (red) from experiments 1, 2, and 3 are shown in Fig. 6a. Reproducibility was evaluated by calculating intra- and inter-assay CVs, summarized in Table 1, along with the corresponding mean and standard deviation values. Acceptable variability was defined as intra-assay CVs below 10% and inter-assay CVs below 15%, with values exceeding these thresholds highlighted in bold. Under Protocol I, multiple CVs exceeded the defined limits at both intra- and inter-assay levels. In contrast, Protocol II generally yielded CVs within the acceptable range, with only one inter-assay CV exceeding the threshold.

**Table 1:** Intra- and inter-assay variability of impedance values using two differentiation protocols –Protocol I and II– in control (Ctl1) and Duchenne (DMD1) cell lines.

		Protocol I					Protocol II				
		Exp 1	Exp 2	Exp 3	Intra CV (Mean)	Inter CV	Exp 1	Exp 2	Exp 3	Intra CV (Mean)	Inter CV
Max Conf	Ctl1 DMD1	96 ± 3.8 122.5 ± 2.6	58.2 ± 2.5 88.8 ± 3	72.6 ± 2.1 79.6 ± 2.7	3.7 3	**25.2** **23.3**	94.5 ± 5.5 136.4 ± 6.6	112.7 ± 2.9 132.6 ± 5.2	91.6 ± 4.7 106 ± 4	4.6 4.2	11.5 13.3
1 dpd	Ctl1 DMD1	66.4 ± 3.3 72.8 ± 2.3	44 ± 2.5 52.3 ± 1.8	47.7 ± 1.4 37.2 ± 4.5	4.5 6.3	**22.7** **33.1**	67.5 ± 3.1 82.1 ± 2.4	69.7 ± 3.1 75.5 ± 3.2	67.8 ± 2.4 71.6 ± 2.6	4.2 3.6	1.7 7
2 dpd	Ctl1 DMD1	49.6 ± 2.9 55.8 ± 2.3	45.7 ± 3.3 44.1 ± 1.6	48.4 ± 2.6 32.5 ± 3.8	6.1 6.5	4.2 **26.4**	65.8 ± 3.5 65.5 ± 1.7	73.8 ± 4 63.9 ± 2.5	66.4. ± 2.2 57.5 ± 2.3	4.7 3.5	6.5 6.8
3 dpd	Ctl1 DMD1	58.2 ± 5.4 68.4 ± 4.6	73.2 ± 5.3 70.2 ± 6.6	57.9 ± 10.8 48.2 ± 5.2	**11.8** 9	13.9 **19.7**	46.1 ± 3.3 53 ± 2	60.3 ± 6.2 62.9 ± 1.8	55.5 ± 4.8 59.9 ± 1.6	8.7 3.1	13.4 8.6
4 dpd	Ctl1 DMD1	35.6 ± 4.2 46.2 ± 2.9	54.2 ± 9.4 46.2 ± 3.9	49.5 ± 8.6 45.6 ± 5.3	**15.4** 8.8	**20.9** 0.7	33.6 ± 2 44.8 ± 2.2	42.1 ± 6.2 56.4 ± 2.3	51.4 ± 2.7 50.8 ± 1.7	8.7 4.1	**21** 11.5

Values are presented as mean ± standard deviation. Intra CV: mean of the coefficient of variation (%) calculated for each experiment. Inter CV: coefficient of variation across experiment means. CV values exceeding the established thresholds, 10 and 15% for intra- and inter-assay CVs, respectively, are highlighted in Bold. Exp, experiment; Max Conf., maximal confluence; dpd, days post differentiation.

Finally, we assessed whether the dystrophic phenotype previously observed under our standard conditions could also be detected using the simplified protocol. Notably, DMD1 cells displayed a greater decline in impedance compared to Ctl1 (Fig. 6b, p<0.05), supporting the suitability of Protocol II for phenotypic assessment. Representative bright-field images of myotubes at 4 dpd in each condition are shown in Fig. 6c.

### Functional evaluation of calcium handling in impedance plates

Our next objective was to determine whether the impedance-based approach is compatible with downstream applications, such as Ca^2+^ handling measurements. To this end, cells were differentiated using Protocol I, which has been extensively validated for its ability to generate functionally mature myotubes at endpoint.

**Fig. 7:**
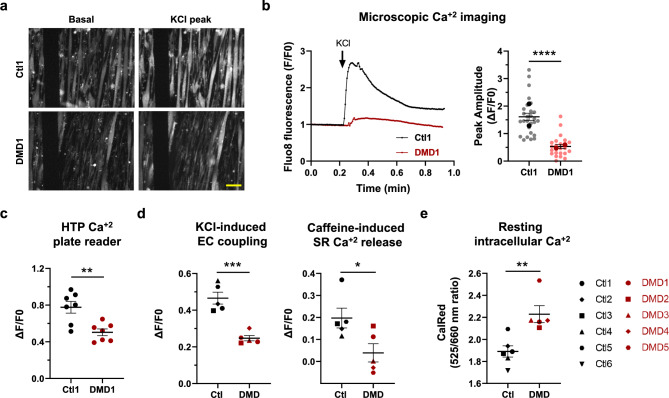
Validation of a high-throughput calcium imaging assay in impedance plates and evaluation of phenotypic alterations in dystrophic myotubes. (**a**) Representative fluorescence images of Fluo-8-loaded control (Ctl1) and dystrophic (DMD1) myotubes at baseline and after KCl stimulation. Scale bar: 100 µm. (**b**) Quantification of calcium (Ca^2+^) release in response to 20 mM KCl by microscopy. Left: Representative traces of normalized Ca^2+^ fluorescence (F/F0). Right: Peak amplitude of Ca^2+^ release (∆F/F0). n = 27 Ctl1 and n = 24 DMD1 myotubes from two independent experiments. Small dots represent individual myotubes; bold dots represent experiment averages. (**c**) High-throughput (HTP) Ca^2+^ imaging using a plate reader: ∆F/F0 after stimulation with 35 mM KCl. n = 7 independent experiments with at least 5 replicates per line. (**d**) ∆F/F0 measured by HTP imaging with Fluo-8 in response to 35 mM KCl (excitation contraction -EC- coupling, left) and 20 mM caffeine (sarcoplasmic reticulum -SR- Ca^2+^ release, right) in control (Ctl, black), and Duchenne (DMD, red) myotubes at 4 days post differentiation (dpd). Data are from n = 5 Ctl and n = 5 dystrophic cell lines performed in 3 replicates. (**e**) Resting intracellular Ca^2+^ levels at 4 dpd measured with the ratiometric indicator CalRed in HTP manner using a plate reader. Data are from n = 6 Ctl and n = 5 dystrophic cell lines from 2 independent experiments performed in 6 replicates. All data expressed as mean ± SEM. *p < 0.05, **p < 0.01, ***p < 0.001, ****p < 0.0001 Unpaired T test.

We first employed fluorescence microscopy-based Ca^2+^ imaging to assess excitation-contraction (EC) coupling triggered by KCl stimulation in Ctl1 and DMD1 lines. Representative Fluo-8 fluorescence images of Ctl1 and DMD1 myotubes before and after KCl addition are shown in Fig. 7a. Quantification of fluorescence in individual myotubes revealed a 68% reduction in EC coupling in DMD1 cells, as indicated by a decreased peak amplitude (∆F/F0) following KCl treatment (Fig. 7b, p<0.0001).

In line with our goal of developing a HTP platform, we next analyzed Ca^2+^ transients using a plate reader that enables rapid data acquisition and processing. This approach revealed a 35% reduction consistent with the microscopy results across seven independent experiments (Fig. 7c, p<0.01). Once validated the compatibility of this technique, we next evaluated EC coupling and sarcoplasmic reticulum (SR) Ca^2+^ content in multiple control and dystrophic lines (Fig. 7d). Consistent with previous results, dystrophic myotubes exhibited a 47% decrease in EC coupling in response to KCl-induced depolarization (p<0.001). Furthermore, caffeine-induced SR Ca^2+^ release was significantly reduced in dystrophic cells compared to controls (80%, p<0.05). Lastly, we measured basal cytosolic Ca^2+^ levels using the ratiometric indicator CalRed with the plate reader. Across two independent experiments, dystrophic myotubes exhibited significantly elevated (18%) resting intracellular Ca^2+^ levels relative to controls (Fig. 7e<0.01).

## Discussion

In this study, we present an impedance-based platform to evaluate the distinct stages of myogenesis, assess the myogenic potential of various ECM coatings, and characterize phenotypic alterations in disease models. While impedance biosensors have been widely used to monitor dynamic physiological changes in adherent cells^19^, their application to human skeletal muscle models remains limited. Most studies to date have focused on murine C2C12 cells^23–25^, and more recently, we demonstrated their potential for identifying myogenic deficits in patient-derived myoblasts^11^. Here, we expanded this previous work by providing a systematic and multiparametric characterization of impedance measurements throughout human myogenesis. By integrating cell morphology, functional readouts, and assay reproducibility, we demonstrate the robustness and limitations of this technique for modeling skeletal muscle development and disease.

We first employed the control 8220 human myoblast line^21,26^, referred to as Ctl1 in this study, to investigate the progression of myogenesis under varying seeding densities: 10k, 20k, and 40k cells/well, corresponding to 500, 1000 and 2000 cells/mm^2^, respectively. During the proliferative phase, we observed a significant positive correlation between impedance values and cell-covered surface area at the lowest seeding density (10k), supporting the utility of impedance for tracking myoblast proliferation^27^. More importantly, our data demonstrate that impedance can also be used to monitor myotube formation and maturation. In line with previous studies using murine models to assess myotube hypertrophy and atrophy in response to insulin-like growth factor-1 (IGF-1) stimulation^23^, our results showed that impedance measurements correlate with myotube thickness in uncoated wells across different seeding densities and time points. When extending this analysis across various coatings, we observed a moderate but significant correlation between impedance and myotube thickness and myotube coverage, and a stronger correlation with total surface coverage. These findings confirm that impedance reliably reflects overall cell-covered surface area and can moderately capture changes in myotube morphology. Nonetheless, since impedance integrates the signal from the entire well, including both myotubes and unfused myoblasts, its ability to accurately reflect myotube atrophy or hypertrophy may be compromised in disease models with impaired fusion, where the presence of unfused cells could mask myotube impedance values. Therefore, while impedance provides valuable insights into overall cell behavior, it is recommended that these measurements be complemented with detailed morphological analyses to accurately interpret myotube structure and health. Despite this limitation, we believe that when comparing conditions within the same cell line, such as untreated versus treated cultures, impedance in uncoated wells can reliably reflect myotube thickness, offering a practical tool for assessing morphological changes in a standardized context.

Seeding density had a strong impact on myotube morphology, with 40k cells/well cultures forming significantly wider myotubes (∼32 µm) than those seeded at 20k or 10k cells/well (∼23 µm and∼18 µm, respectively). These values are comparable to those reported under pulsed electromagnetic field stimulation^28^, suggesting that this system may enhance myotube maturation similarly. Notably, commonly used protocols with standard plates typically yield thinner myotubes (∼15 µm)^29–31^. The use of IGF-1-enriched medium (10 ng/mL) in our protocol, a known promyogenic and hypertrophic factor, likely contributed to these results, consistent with previous work^15^. Interestingly, myoblasts seeded in 96-well impedance Z-plates exhibited a remarkable spontaneous alignment along the electrode axis, even in the absence of pre-patterned substrates. Also, the weak alternating current applied during real-time impedance recordings appeared to further enhance this alignment, reducing the random orientation typically seen in conventional 2D cultures^32^. Despite enhanced alignment, no significant differences were observed in the FI. However, the reduced coefficient of variation observed in Z-plates compared to standard plates suggests a greater reproducibility, which is a critical feature for disease modeling and HTP screening applications. Z-plates supported the formation of more elongated myotubes, with fewer branching points, features that have been associated with enhanced myotube maturation and contractile function in previous studies^33^. These features, together with the higher myotube alignment observed in Z-plates, may reflect a shift toward more mature and less heterogeneous myotube cultures. We believe that this analysis adds a novel dimension to the interpretation of impedance-based alignment and supports the utility of the platform for generating higher quality myotube cultures and studying subtle morphological phenotypes. A limitation of the present study is that Z-plates, while highly suitable for real-time monitoring, are not optimal for high-resolution microscopy. This constraint prevented us from reliably quantifying cell–cell junction integrity or assessing sarcomere striation patterns in α-actinin–positive myotubes, both of which would have provided additional insight into the structural basis of the impedance signal. Nevertheless, these analyses could be implemented in future studies using imaging-compatible substrates. Importantly, the main advantage of the impedance platform is that it delivers continuous, non-invasive measurements of culture dynamics, in contrast to conventional end-point assays that require destructive processing and provide only static snapshots.

Building on these findings, and with the aim of further optimizing culture conditions for robust impedance-based myogenesis assessment, we next sought to explore whether modifying the culture substrate could influence key aspects of the myogenic process, including adhesion, proliferation, differentiation and maturation. Given that ECM components play a critical role in muscle development and regeneration, we compared the myogenic potential in different substrate coatings, including commonly used coatings, such as gelatin and ECM-Gel, and two decellularized tissue-derived matrices: FATRIX, from adipose tissue, and MATRIX, from skeletal muscle tissue. ECM-Gel significantly improved myoblast attachment compared to other coatings, likely due to its composition that includes components of ECM such as laminin and type IV collagen, proteins well known to promote cell adhesion and migration^2^. However, enhanced adhesion in ECM coatings did not translate into increased proliferation. In fact, uncoated wells supported the highest proliferation rates, reaching confluence sooner and displaying the greatest impedance at this stage, suggesting that Z-plates may offer a favorable environment for early cell expansion. Uncoated wells also showed increased myotube thickness at early maturation stage (5 dpd); however, this effect was transient and by 7 dpd uncoated wells presented a marked decline in myotube thickness. Notably, only MATRIX-coating preserved myotube thickness between 5 and 7 dpd, as well as impedance values and total and myotube surface coverage. These results suggest that the muscle-derived coating provides essential cues to support long-term myotube maturation and counteract atrophic progression, likely due to the presence of muscle-specific ECM components^34^. In contrast, FATRIX did not prevent the progressive atrophy of myotubes observed during the culture lifespan, suggesting that it lacks these muscle-specific signals required to sustain myotube homeostasis. Alternatively, the reduced biological activity of FATRIX may be partly explained by differences in its processing, as, unlike MATRIX, it did not undergo a pepsin digestion step, which may enrich for bioactive ECM components. In any case, FATRIX may offer a relevant microenvironment to model diseases characterized by fat infiltration into skeletal muscle, such as DMD or sarcopenia^35^.

Given the well-documented deficits in proliferation, migration, and differentiation of DMD myoblasts^8,29,36–38^, impedance-based approaches offer a promising avenue to monitor these processes in real time and under physiologically relevant conditions. Initial characterization of myotube maturation, based on MyHC expression and FI, revealed no significant differences between control and dystrophic cells. This contrasts with previous studies that have reported impaired fusion in DMD cells^38–40^. However, other reports have shown comparable fusion in dystrophic myotubes derived from patient fibroblasts, iPSC-derived myoblasts, or DMD myoblasts^41,42^, suggesting that differences in differentiation protocols or confluency levels, such as the supra-confluent conditions reached in our study, may influence these outcomes and potentially mask subtle fusion deficits^42^.

Beyond fusion, impedance measurements allowed us to detect early alterations in dystrophic cultures that were not evident through conventional morphological markers. Notably, increased impedance values at early time points correlated with higher cell numbers in DMD cultures, consistent with reports of altered cell cycle regulation and hyperproliferation in dystrophic myoblasts^35^. However, this initial proliferative advantage did not translate into improved differentiation, as dystrophic cultures exhibited reduced baseline-corrected impedance values (relative to 0 dpd) during early differentiation and maturation. Importantly, dystrophic myotubes also exhibited significantly reduced width and myotube coverage. These findings align with previous observations of impaired survival and maturation in DMD myotubes^20,29,36–38^, and underscore the multifactorial nature of dystrophic pathology. Importantly, our data support the utility of impedance-based analysis to detect dystrophic myogenic phenotypes. In fact, we previously observed similar abnormalities in Myotonic Dystrophy type 1 using impedance-based measurements^11^, further supporting the relevance of this technique for investigating disease-associated defects. Nonetheless, our data also highlight the importance of complementing impedance measurements with detailed morphological analyses to fully capture the complexity of myogenic progression, particularly in models with impaired fusion or increased cell death. The use of five patient-derived lines in this study, encompassing three distinct DMD deletions and originating from different muscle types, reflects the biological diversity typically encountered in human cohorts. While this heterogeneity may contribute to variability in the data, it also enhances the translational relevance of the findings by capturing a broader spectrum of dystrophic phenotypes. Our focus was to identify consistent trends across dystrophic lines and to demonstrate the sensitivity of impedance-based measurements in detecting disease-relevant alterations despite inter-patient variability. Future studies incorporating larger cohorts will be essential to dissect genotype- and tissue-specific contributions to the observed phenotypes.

To further refine our approach, we examined the impact of two differentiation protocols, as protocol selection plays a critical role in both assay reproducibility and phenotypic resolution. We compared our standard differentiation protocol (Protocol I)^20,21^ with a simplified, literature-based approach (Protocol II)^22^ to determine their effect on the consistency of the impedance profile. Both protocols effectively supported the formation of mature myotubes across representative cell lines. However, unlike Protocol I, Protocol II consistently maintained impedance variability within established thresholds (below 10% for intra-assay and 15% for inter-assay coefficients of variation)^43^, highlighting its superior reproducibility. These findings are particularly relevant for HTP and disease modeling applications, where minimizing technical variability is essential.

Beyond structural and impedance-based assessments, we explored Ca^2+^ dynamics as a complementary functional readout of myotube maturation and disease-associated phenotypes. A key advantage of the impedance-compatible platform used in this study is its suitability for downstream HTP functional assays, such as Ca^2+^ imaging in 96-well formats. In this sense, here, we used the fluorescent Ca^2+^ indicator Fluo-8 AM to evaluate KCl-induced Ca^2+^ transients via microscoy and plate reader-based assays in control (Ctl1) and dystrophic (DMD1) myotubes. Both approaches revealed the expected reduction in Ca^2+^ peak amplitude in dystrophic cells upon depolarization, in line with previous reports of impaired EC coupling in DMD muscle^44–47^. However, the amplitude differences were more pronounced in microscopy-based measurements, likely reflecting the confounding contribution of unfused myoblasts in the bulk signal acquired by the plate reader. This highlights the importance of considering cellular heterogeneity and myogenic stage when interpreting population-level functional assays. Nevertheless, the plate reader approach proved sufficiently sensitive and reproducible to detect consistent KCl response deficits across multiple dystrophic lines, reinforcing its value for phenotypic screening applications.

To further dissect intracellular Ca^2+^ dynamics in the HTP platform, we used caffeine stimulation to evaluate SR Ca^2+^ content and the ratiometric indicator CalRed to evaluate resting cytosolic Ca^2+^ levels in myotubes from control and dystrophic patients. Dystrophic myotubes showed significantly blunted Ca^2+^ release in response to caffeine, consistent with previous studies demonstrating RyR1 dysfunction and SR Ca^2+^ depletion in DMD patient-derived cells and *mdx* mouse models^38,48,49^. In addition, elevated intracellular Ca^2+^ was consistently observed in dystrophic myotubes, corroborating earlier findings in DMD muscle cells and tissues^20,38,48,50–55^. Notably, these increases were predominantly detected in contractile myotubes from dystrophic patients, supporting the idea that abnormal Ca^2+^ accumulation in DMD muscle is triggered or exacerbated by contractile activity^56–58^.

In summary, our findings establish impedance-based monitoring as a robust and sensitive method for tracking human myogenesis *in vitro*. The strong correlation between impedance signals and key morphological parameters and seeding density, underscores its value as a non-invasive surrogate marker of myogenic progression. Furthermore, the platform’s compatibility with functional readouts, including both microscopy- and plate-based live Ca^2+^ imaging, expands its utility beyond structural assessment. Notably, the system’s high reproducibility, scalability, and real-time monitoring capabilities make it particularly well-suited for HTP screening and the evaluation of biomaterials or pharmacological interventions. As such, this integrated approach offers a powerful and versatile framework for phenotypic characterization in neuromuscular disease models, with strong potential for both basic research and translational applications. While the present study focused on the technical and biological validation of the platform, future work will include proof-of-concept pharmacological rescue experiments using known therapeutic compounds, such as TGF-β inhibitors or utrophin upregulators, to further demonstrate the utility of impedance and Ca^2+^ imaging readouts for therapeutic screening in dystrophic models.

## Methods

### Cell cultures

In this study, we used a total of 11 human immortalized myoblasts lines (Supplementary Table S1): six from healthy control donors, and five from patients with Duchenne muscular dystrophy (DMD).

Most cell lines were generated by the Platform for Immortalization of Human Cells (Myology Institute, Paris) and were kindly provided by Dr. Vincent Mouly, Dr. Virginia Arechavala, Dr. Gisela Nogales-Gadea, and Dr Amets Sáenz. The use of these cell lines was approved by the respective institutional ethics committees at the source institutions and by the Ethical Committee of the Basque Country.

### Culture conditions

In this study, we employed two distinct myogenic differentiation protocols. Protocol I corresponds to our in-house standard, based on previously described procedures^20,21^. Briefly, cells were cultured in Skeletal muscle cell Growth Medium (SGM) until confluence, at which point the medium was replaced with basic differentiation medium (bDM). As myoblast fusion progressed and multinucleated myotubes became evident, bDM was substituted with complete differentiation medium (cDM) to support further maturation. Protocol II follows a literature-based approach^22^ that employs a single basal medium throughout both proliferation and differentiation stages. Initially, cells were cultured in Ultroser proliferation medium, which was replaced the next day with human epidermal growth factor differentiation medium (hEGF-DM) to induce myogenesis and promote maturation. In both protocols, half of the culture medium was refreshed every two days. Supplementary Table S2 includes the composition of used media.

### Extracellular matrix and coatings

We evaluated the myogenic potential of porcine-derived decellularized adipose tissue (FATRIX) and skeletal muscle (MATRIX), in comparison with the positive controls gelatin (Sigma, G1890) and ECM-Gel (Sigma, E1270). Uncoated wells were used as negative control. Gelatin was prepared at 0.5% (w/v) in water, while ECM-Gel, FATRIX, and MATRIX were diluted 1:100 in DMEM. All coatings were incubated for 30 minutes at 37ºC and 5% CO2.

- FATRIX preparation: FATRIX was prepared as previously described^59^. Briefly, porcine adipose tissue was obtained from a local food supplier (JAUCHA S.L., Navarra, Spain). The tissue was cleaned, mechanically creamed using a beater, and stored at -20 °C. For decellularization, protein pellets were isolated by homogenizing the tissue with a Polytron homogenizer (PT3100) at 12,000 rpm for 5 min, followed by a centrifugation at 5000 rpm for 5 min in ultrapure water. Lipid layers were manually removed, and the remaining protein pellets were sequentially incubated at room temperature in an orbital shaker at 100 rpm overnight with isopropanol and for 36 h with 1% (v/v) Triton X-100 in 0.1% (v/v) ammonium hydroxide (all reagents from Merck Life Science). Each step was followed by thorough washing with Phosphate Buffer Saline (PBS, Merck) supplemented with 1% (v/v) antibiotic antimycotic solution (Gibco-BRL, Paisley, UK) and 125 µL protease inhibitors (539,128, Merck Life Science), and a final rinse with Ultrapure Milli-Q water. The resulting material was dried and milled into a fine powder using a mixer mill (Retsch MM400, Haan, Germany) and stored at 4 °C in a vacuum desiccator. The resulting powder was dissolved at a concentration of 2 mg/mL in 1 M acetic acid (Panreac Quimica SLU, Barcelona, Spain) under continuous stirring for 48 h at room temperature. Decellualrization criteria, including DNA quantification and absence of nuclear material, were confirmed as previously described^59^. Additionally, a detailed proteomic characterization of the FATRIX material was reported in the same study.
- MATRIX preparation: *Biceps femoris* muscle samples were collected postmortem from 2-month-old female Large White pigs employed for surgical training at the BioGipuzkoa Health Research Institute animal facility. After dissection and removal of surrounding tissue, the samples were cut into 1 cm^2^ pieces and stored at  − 8 0 °C until further processing. For the decellularization, a custom protocol was developed following the methodology outlined by Crapo et al^60^. Briefly, samples underwent a freeze–thaw cycle and were then treated overnight with a hypertonic solution containing 1.5 M sodium chloride, 1% ethylenediaminetetraacetic acid (EDTA)(v/v), and 10 mM Tris. Subsequently, all tissues were incubated in a detergent solution containing 1% sodium dodecyl sulfate and 1% sodium deoxycholate (v/v) for 48 h. Following detergent treatment, tissues were subjected to overnight nuclease digestion (DNARASE, c-LEcta) (40 U/ml) and then immersed in a 50 mM Tris/1.5 M NaCl-based hypertonic solution for 4 h. Finally, sterilization was performed using a combination of 4% ethanol and 0.1% peracetic acid (v/v) for 2 h, followed by a 72-h wash in sterile PBS. The resulting decellularized ECMs were lyophilized and milled using a Pulverisette 14 mill (Fritsch) at 10,000 rpm. The resulting powder was digested at a concentration of 2 mg/mL in a solution of 0.5 M acetic acid (Sigma) and 1 mg/mL pepsin (Sigma) under agitation at room temperature for 48 h. After digestion, the pH was adjusted to a range of 7.4–7.6, and the samples were stored at 4 °C until further use.

### Impedance measurements

Impedance analyses were performed using CytoView-Z 96 plates (Axion Biosystems, Z96-IMP-96B), hereafter referred to as Z-plates. Each well contains a single interdigitated gold electrode (100 µm width and spacing) embedded in a polyethylene terephthalate substrate, with a total recording area of 20 mm^2^. Prior to cell seeding, plates were coated with the previously described ECM materials or left uncoated. After removal of the coating solution, 100 µL of proliferation medium was added to each well, and baseline impedance was recorded using the Impedance Module of Maestro Edge (Axion Biosystems). Subsequently, 100 µL of cell suspension was added to each well to reach final seeding densities of 10,000 (10k), 20,000 (20k), or 40,000 (40k) cells/well. Plates were incubated at room temperature for 1 hour to ensure uniform cell distribution across the surface. Continuous impedance monitoring was then performed in the Maestro Edge at 37 ºC with 5% CO2. Resistance values were acquired every minute at 41.5 kHz using AxIS Z software (Version 3.12.1.10; https://www.axionbiosystems.com). To evaluate the dystrophic phenotype, impedance values were baseline-corrected by subtracting the value recorded at 0 days post differentiation (0 dpd).

### Total coverage, myotube coverage, myotube thickness, and alignment

High-resolution bright-field images were acquired using an ECLIPSE Ti-S/L100 inverted microscope (Nikon) equipped with a 10x Plan Fluor objective, a lambda-DG4 illumination system, and an Orca-Flash2.8 camera (Hamamatsu). Image acquisition was performed using Nis-Elements software (Advanced Research, Version 5.11.03; https://www.microscope.healthcare.nikon.com). Image analysis was carried out in ImageJ (Version 2.9.0; https://imagej.net/software/fiji). Total coverage was assessed using the PHANTAST segmentation plugin. Myotube coverage was measured by manual tracing of individual cells. Myotube thickness was quantified by calculating the mean diameter of individual myotubes. Alignment analysis was performed using the OrientationJ Distribution plugin, based on histograms of orientation angles. For experiments comparing different seeding densities or ECM coatings, three fields of view (FOVs) per well and four wells per condition were analyzed. For the morphological evaluation in control and dystrophic lines, one FOV from five different wells per line was used for myotube thickness analysis, and one FOV from one well per line for myotube coverage analysis.

### Automated capillary Western blot

Protein was extracted from cell pellets using RIPA lysis buffer (50 mM Tris-HCl pH 7.2, 0.9% NaCl, 1% NP40, 1 mM EGTA, 1 mM EDTA) containing protease and phosphatase inhibitors (Thermo Fisher, 748443) and inhibitors of calpain I/II and cathepsins B/L (Sigma, 208719). Protein quantification was performed by the Bradford protein assay (Bio-Rad, 500-0006). Capillary Western blot was performed using the Jess instrument (ProteinSimple; Biotechne, Minneapolis, MN, USA), following the manufacturer’s instructions. Protein samples were diluted to 0.5 µg/µL with 5X Fluorescence Master Mix (Biotechne) and denatured at 95 °C for 5 mins. Samples were loaded onto customary 66-440 kDa separation module plates (Biotechne, SM-W005) for protein separation. Used primary antibodies were anti-dystrophin MANDYS1 (Clone 3B7, DSHB) and anti-myosin heavy chain (MyH1, clone A4.1025, DSHB), both diluted 1:50 in Antibody Diluent 2. Chemiluminescence signals were quantified using the Compass for SW software (Version 6.0.0; https://www.proteinsimple.com), which generated chemiluminescence spectra and lane view images. Signals were normalized to total protein (Biotechne, DM-TP01) and represented as fold change versus controls.

### Immunofluorescence and fusion index

To evaluate the impact of plate substrate and impedance-monitoring current application on myogenic differentiation, Ctl1 control myoblasts at 5 dpd cultured in a standard 96-well plate, a Z-plate without current, or a Z-plate with current were fixed with 4% paraformaldehyde for 10 min and incubated with a blocking solution (2% BSA, 1% GS, 0.5% triton X-100 and 0.02% NaN3 in PBS) for 1 hour at room temperature. For myotube visualization anti-*α* actinin mouse mAb (1:200, A7811 Sigma-Aldrich) was used and incubated overnight at 4 °C. After washing, cells were incubated with Alexa Fluor 555-conjugated secondary antibody for 1h at room temperature. Nuclei were stained with DAPI (Sigma-Aldrich, D9542), and samples were mounted using ProLong™ Diamond Antifade Mountant (Thermo Fisher Scientific, P36962). Cells were imaged using a ZEISS LS900 confocal microscope. The fusion index was calculated as the percentage of nuclei inside *α*-actinin-positive myotubes. At least two FOVs per well and 4 wells per condition were analyzed. Confocal images were processed using ZEN software (Version 3.0 Blue edition; https://www.zeiss.com/microscopy).

To evaluate the maturation of dystrophic myoblasts, control and dystrophic cell lines were seeded in 0.5% gelatin-coated µ-Slide 15 Well 3D plates (Ibidi, 81506). At 5 dpd, cells were fixed and blocked as described above. For myotube visualization, FITC-conjugated anti-human Myosin Heavy Chain antibody (1:50, IC4470F, R&D) was used and incubated for 1h at room temperature. Following washing, DAPI staining and mounting were performed as described. High-resolution confocal images were acquired with a ZEISS LSM980 microscope equiped with Airyscan2 and a 10x objective (Carl Zeiss). The fusion index was calculated as the percentage of nuclei inside Myosin Heavy Chain-positive myotubes, analyzing three FOVs per cell line.

### Length-to-width ratio and branching points

Length-to-width (L/W) ratio and the number of branching points per myotube were measured in *α-*actinin and DAPI stained myotubes cultured in a standard 96-well plate, a Z-plate without current, or a Z-plate with current. L/W ratio was quantified by manual analysis using ImageJ. Branching points were estimated using the Myotube Analyzer tool^61^.

### Cell number and viability assay

Cell number and viability were assessed using a combination of the ReadyProbes™ Cell Viability Imaging Kit (Blue/Green, R37609, Molecular Probes) and CellTrace™ Calcein Red-Orange AM (C34851, Molecular Probes). Cells were simultaneously stained with 2 µM Calcein Red-Orange AM and two drops per milliliter of medium of NucBlue Live and NucGreen Dead reagents from ReadyProbes™ Cell Viability Imaging Kit, following the manufacturer’s instructions. Staining was performed by incubating the cultures at 37 °C and 5% CO₂ for 15 min. Fluorescence images were acquired using an ECLIPSE Ti-S/L100 microscope (Nikon) equipped with a 10× Plan Fluor objective. Dead nuclei were identified by co-localization of NucGreen signal with Hoechst 33342 (NucBlue) staining. Quantification was performed using ImageJ, and results were expressed as the percentage of dead nuclei relative to total nuclei per field. Calcein staining was used to quantify total coverage and myotube coverage using ImageJ.

### Calcium imaging

Resting cytosolic calcium levels were determined in a HTP format using the ratiometric indicator CalRed™ R525/650 AM (AAT Bioquest, 20591) and the Glomax Discover Microplate Reader (Promega). Human myotubes at 4 dpd were incubated with 4 µM CalRed and 2.5 mM ReadiUse^TM^ Probenecid (AAT Bioquest, 20062), which contains 0.02% Pluronic F-127, in culture medium for 30 min at 37 ºC. After a 15-minute wash with Ringer solution (125 mM NaCl, 5 mM KCl, 1.2 mM MgSO4, 6 mM glucose, 2 mM CaCl2 and 25 mM HEPES, pH 7.4) at room temperature, baseline cytosolic Ca^2+^ levels were recorded with excitation at 475 nm and dual emissions at 500-550 and 660-720 nm. The 525/660 ratio was used for quantification.

To assess Ca^2+^ transients upon stimulation, cells at 4 dpd were incubated with 4 µM Fluo-8-AM (AAT Bioquest, 21082) and 2.5 mM ReadiUse^TM^ Probenecid for 30 min at 37 °C, followed by a 15-minute wash with Ringer solution at room temperature. For microscopy-based imaging, fluorescence (excitation at 488 nm) was recorded using an ECLIPSE Ti-S/L100 microscope (Nikon) equipped with a 10× Plan Fluor objective before and after the addition of 20 mM KCl. For HTP imaging, fluorescence (excitation at 475 nm, emission 500–550 nm) was measured using the Glomax reader before and after stimulation with 20 mM caffeine or 35 mM KCl. Ca^2+^ transients were analyzed as the Peak amplitude (∆F/F0) for each condition.

### Data analysis and statistical procedures

Statistical analyses were performed using GraphPad Prism (Version 8.3.0; https://www.graphpad.com). Outlier values were identified and removed using the ROUT method with Q=0.5%. Data distribution was assessed using the Shapiro–Wilk normality test. Parametric tests were applied to datasets with Gaussian distribution. An Unpaired T-test was used to compare independent groups. For repeated measures at different time points, a Paired t-test was used. One-way ANOVA followed by Dunnett’s or Tukey’s post hoc test was applied for comparisons involving more than two groups. For datasets that did not meet the assumptions of normality, non-parametric tests were applied. Specifically, the Kruskal–Wallis test followed by Dunn’s post hoc multiple comparison test was used for group comparisons. For multi-factorial experiments, a Two-way ANOVA was applied. When appropriate, post hoc tests (Dunnett’s) were used to identify specific group differences. Pearson correlation coefficients were calculated for correlation analyses. For all statistical analyses, adjusted p-values less than 0.05 were considered statistically significant. The coefficient of variation was used to assess reproducibility and was calculated as the percentage ratio of the standard deviation to the mean.

## Supplementary Information

Supplementary Information.

## Data Availability

The datasets used and/or analyzed during the current study are available from the corresponding author on reasonable request.
