# Supplementary material for: Validation of an in vitro muscle platform to evaluate myogenesis and calcium handling in control and dystrophic human myotubes
Source: Sci Rep. 2025 Dec 17;16:1891. doi: 10.1038/s41598-025-31522-z (PMC12804846; doi:10.1038/s41598-025-31522-z)
Supplement: Supplementary file 1 — Supplementary Information. [file 41598_2025_31522_MOESM1_ESM.pdf]

Validation of an *in vitro* muscle platform to evaluate myogenesis and calcium handling in control and dystrophic human myotubes

Laura Mosqueira-Martín, Carolina Prendes-García, Camila Vesga-Castro, Pablo Marco-Moreno, Ainhoa Irastorza, Ander Izeta, Iratxe Madarieta, Itxaso Martí-Carrera, Jacobo Paredes, Adolfo López de Munain, Ainara Vallejo-Illarramendi

Supplementary information

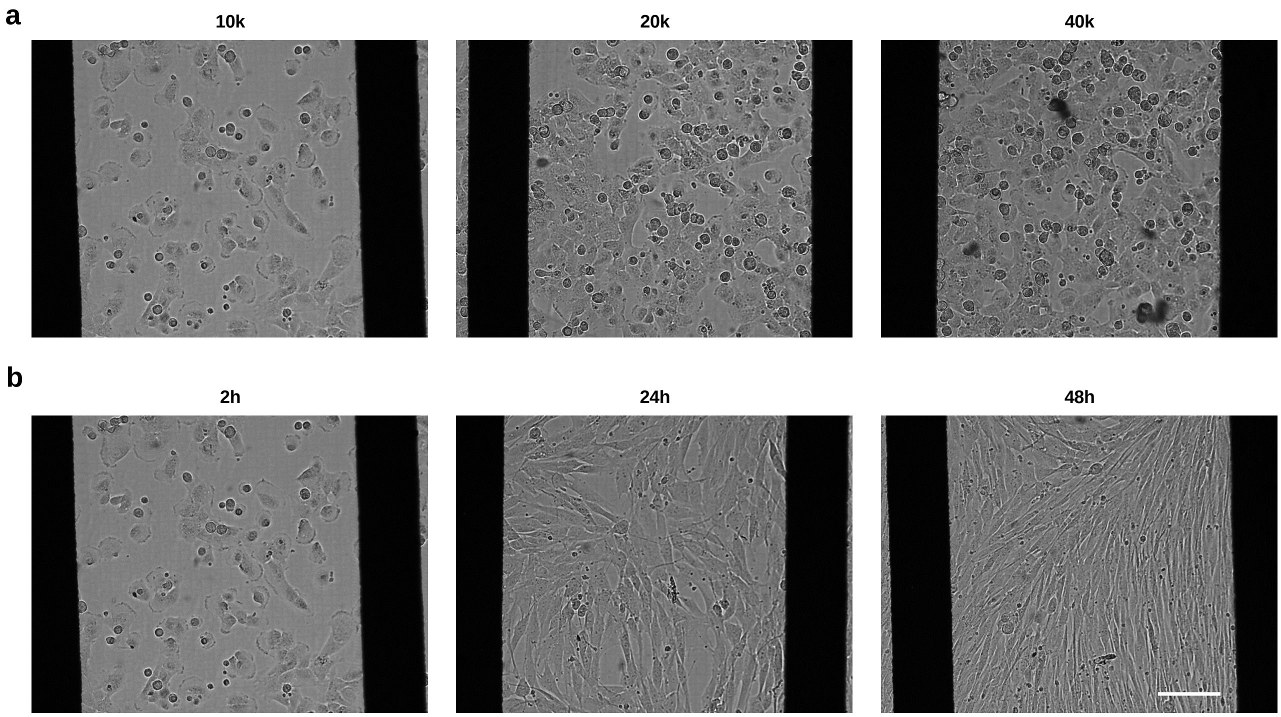

Supplementary Fig. S1. Representative bright-field images of control Ctl1 myoblasts seeded in impedance uncoated plates. (a) Images from cells seeded at 10,000 (10k), 20,000 (20k), and 40,000 (40k) cells per well 2 hours post-seeding. (b) Images from cells seeded at 10k cells/well 2-, 24-, and 48-hours post-seeding. Scale bar: 100 µm.

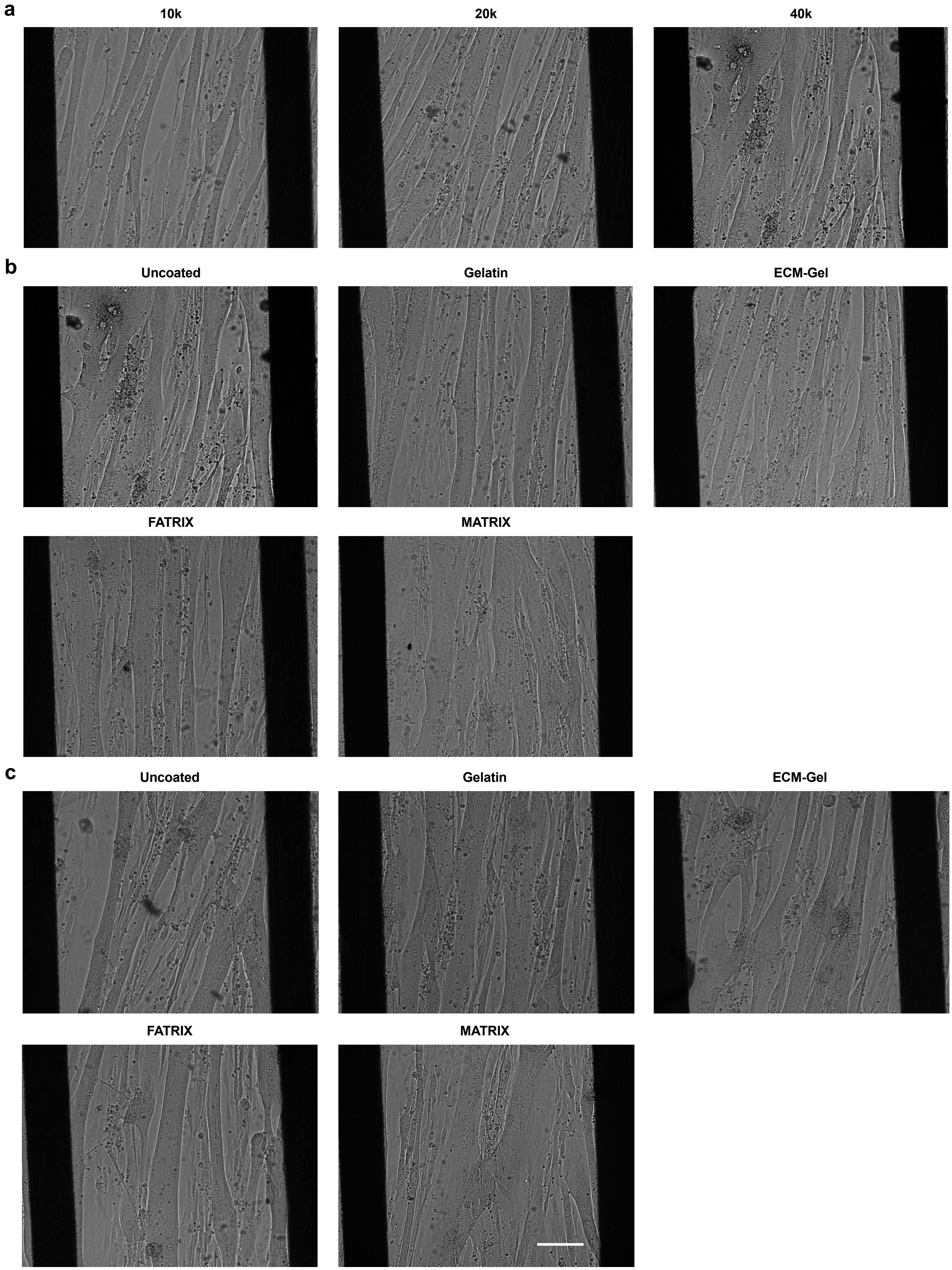

Supplementary Fig. S2. Representative bright-field images of myotubes formed in impedance plates from control Ctl1 cells. (a) Images from cells seeded at 10,000 (10k), 20,000 (20k), and 40,000 (40k) cells per well in uncoated plates at day 5 post differentiation (5dpd). (b-c) Images from cells seeded at 40k cells/well in uncoated plates or plates coated with gelatin, ECM-Gel, FATRIX, or MATRIX at 5 dpd (b) and 7 dpd (c). Scale bar: 100 μm.

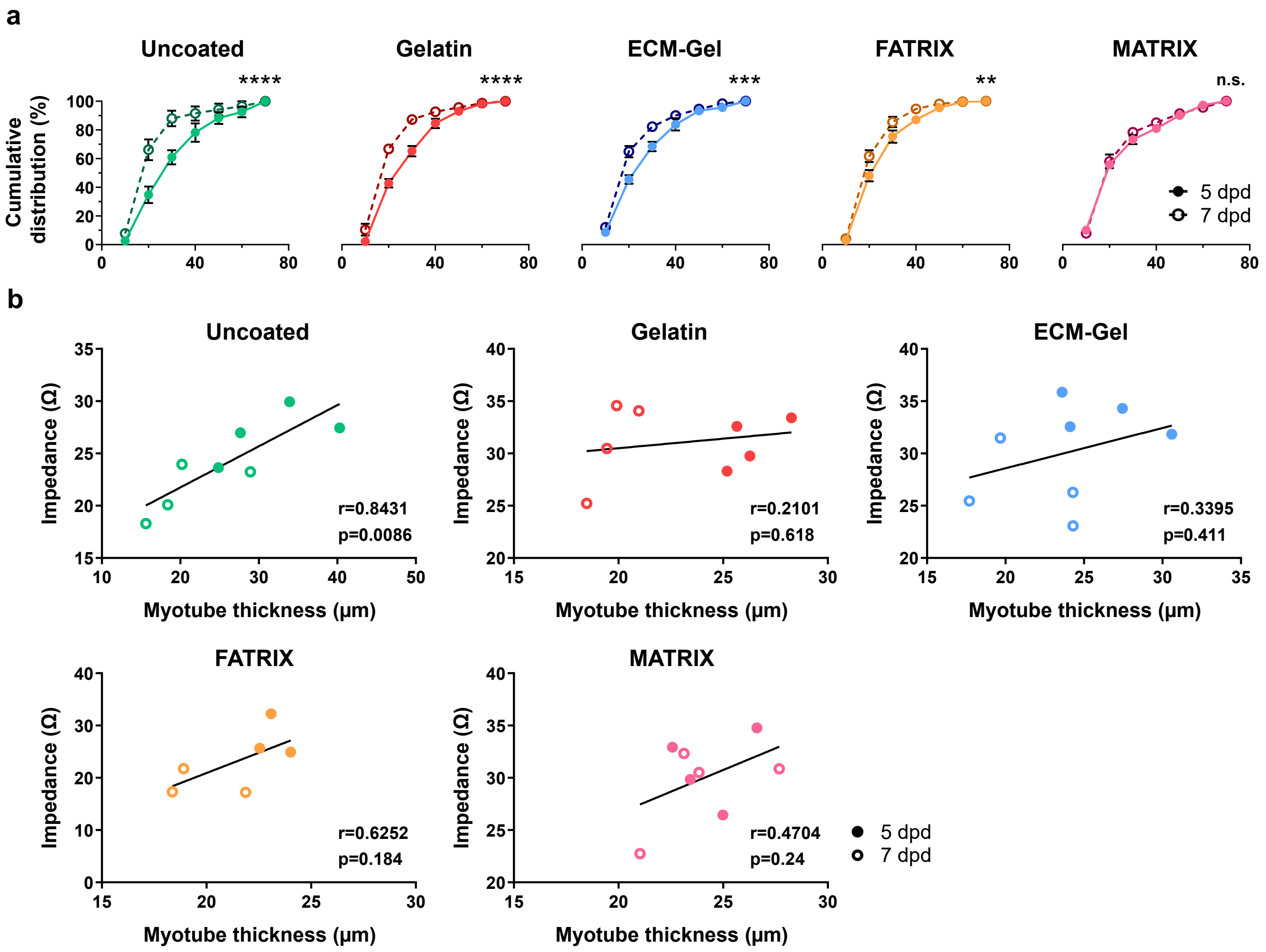

Supplementary Fig. S3. Analysis of myotube diameter distribution and correlation with impedance across coating conditions. (a) Cumulative distribution of myotube diameters at 5 and 7 days in differentiation (dpd) across all coating conditions. Data are expressed as mean ± SEM. $^{**}p<0.01$, $^{***}p<0.001$, $^{****}p<0.0001$, n.s., non significant, Two-way ANOVA. (b) Correlation analysis between myotube thickness and impedance at 5 and 7 dpd. r, Pearson correlation coefficient.

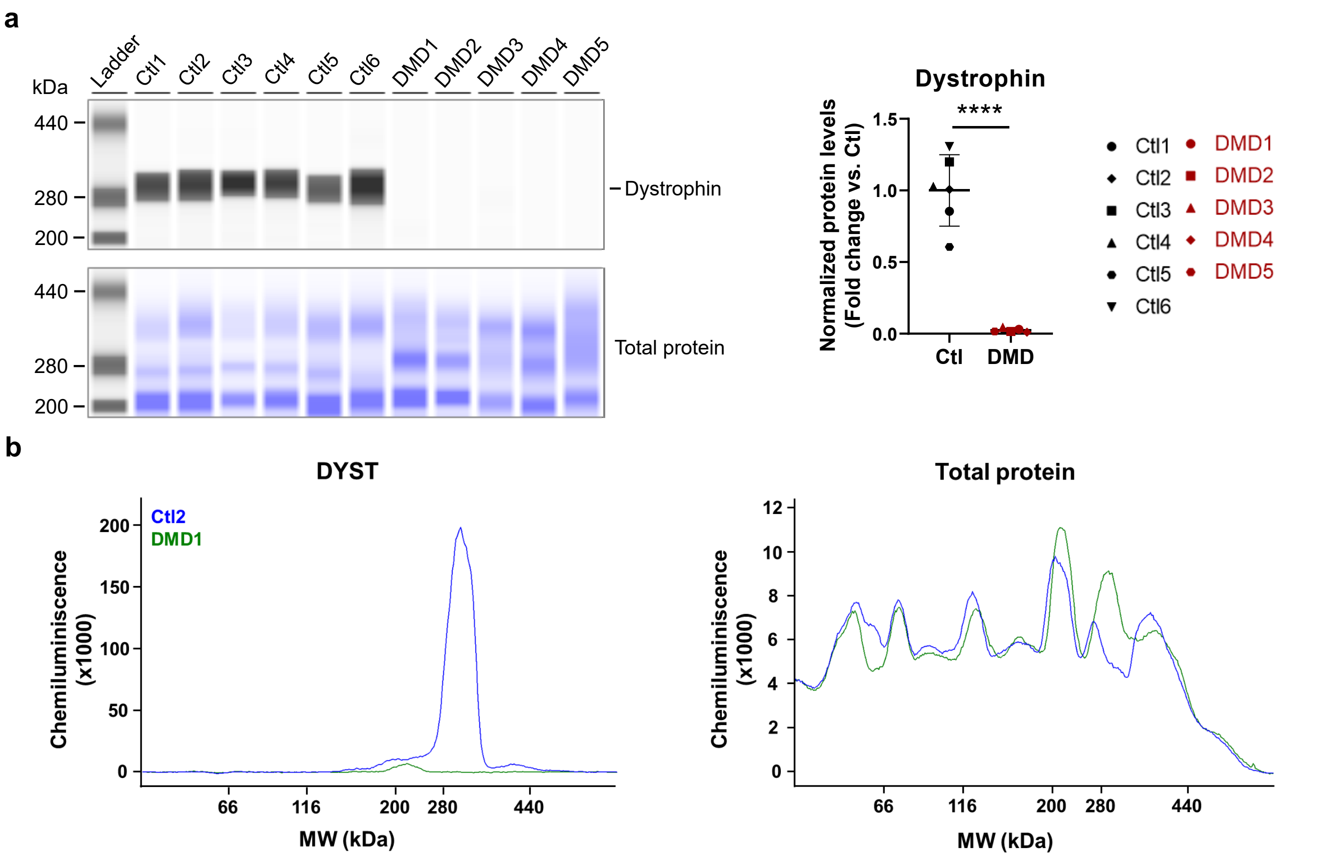

Supplementary Fig. S4. Protein expression of dystrophin in myotubes assessed by capillary western blot. (a) Left: Representative artificial lane view images showing Dystrophin (top) and Total protein (bottom) signals in myotubes derived from control (Ctl), and Duchenne (DMD) cell lines. Right: Quantification of dystrophin levels normalized to total protein and represented as fold change versus control. n=6 Ctl lines (black dots), and n=5 DMD lines (red dots). Data are expressed as mean ± SEM. ****p<0.0001, Unpaired T test. (b) Representative electropherograms showing dystrophin (left) and total protein (right) signals for one control line (Ctl2, blue), and one DMD line (DMD1, green).

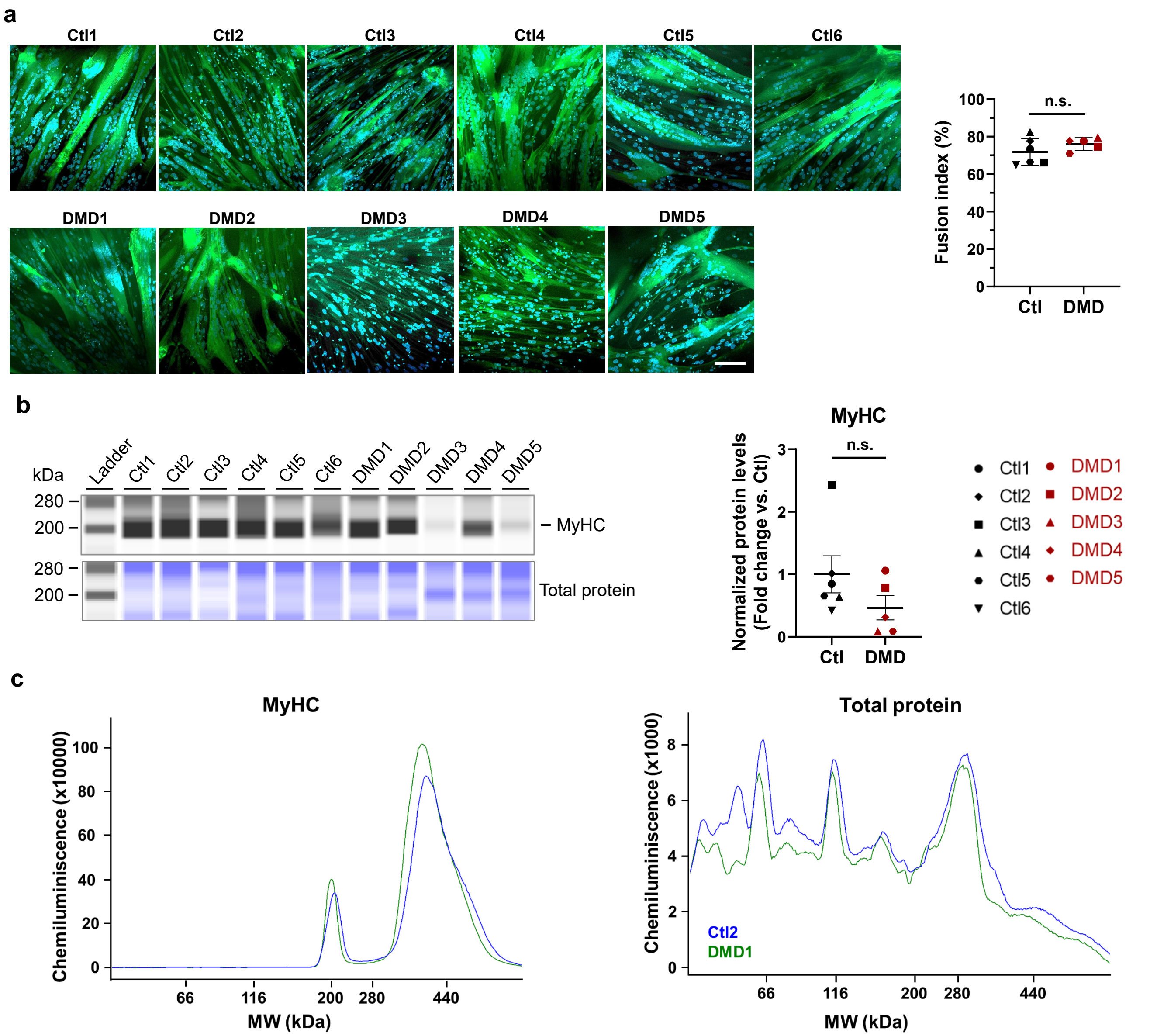

Supplementary Fig. S5. Fusion index and myosin expression in control and dystrophic myotubes. (a) Fusion index at 5 days in differentiation. Left: representative immunofluorescence images stained for Myosin (green) and DAPI (blue). Scale bar: 50 µm. Right: quantification of fusion index (%). (b) Protein expression of myosin heavy chain (MyHC) analyzed by capillary western blot. Left: artificial lane view images of MyHC (top) and Total protein signals (bottom) in myotubes derived from control (Ctl), and Duchenne (DMD) cell lines. Right: quantification of MyHC levels normalized to total protein and represented as fold change versus control. (c) Representative electropherograms of MyHC (left) and total protein (right) signals for one control line (Ctl2, blue), and one DMD line (DMD1, green). Data in (a) and (b) are expressed as mean ± SEM. n=6 Ctl lines (black dots), and n=5 DMD lines (red dots). n.s., non significant, Unpaired T Test.

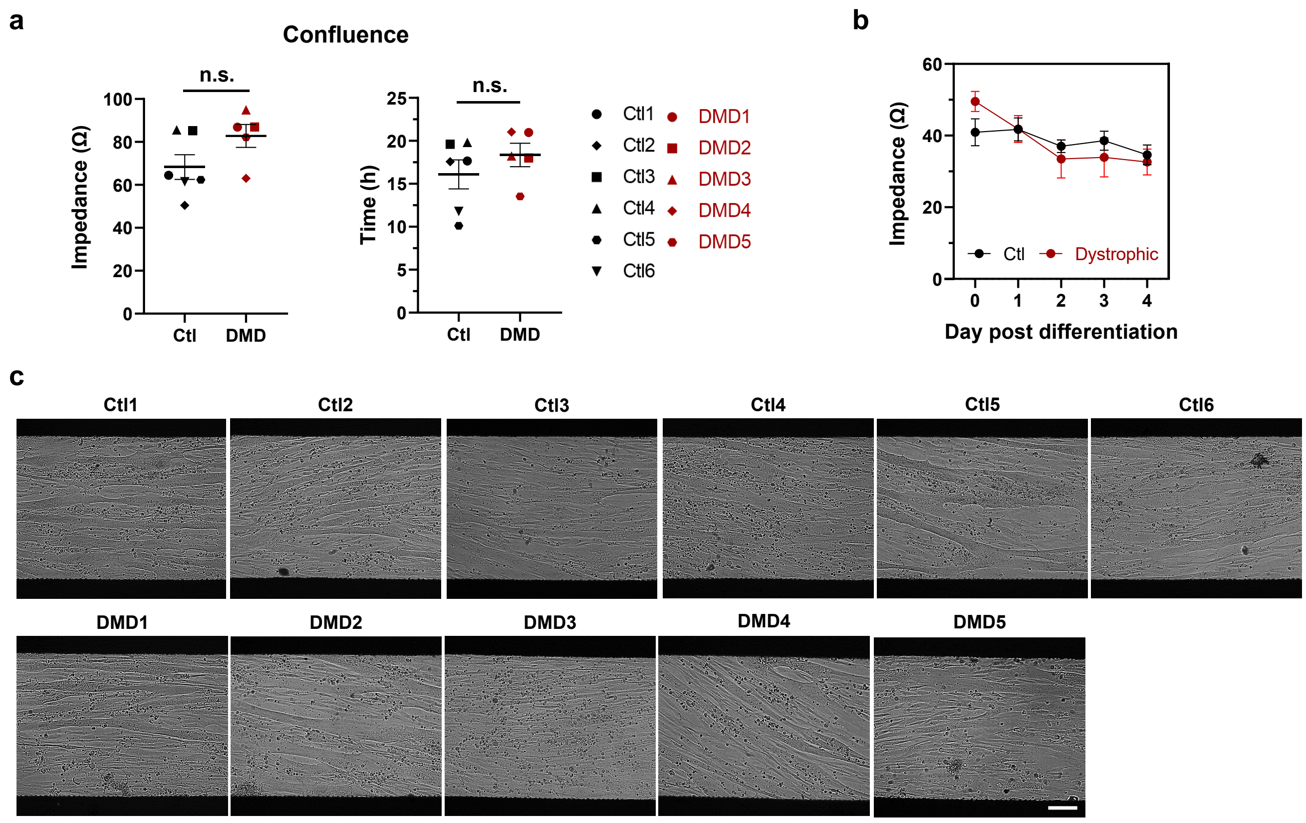

Supplementary Fig. S6. Myogenic features of control and dystrophic myoblasts cultured in impedance plates. (a) Left: Impedance values (Ω) at confluence. Right: Time to reach confluence measured by impedance. (b) Raw impedance values (Ω) from 0 to 4 days in differentiation. (c) Representative bright-field images of myotubes formed in impedance plates from control (Ctl), and Duchenne (DMD) cell lines. Scale bar: 100 µm. All data expressed as mean ± SEM. Data are from n=6 Ctl and n=5 dystrophic cell lines from 3 independent experiments performed in 6 replicates. n.s., non significant, Unpaired T Test.

Supplementary Table S1. General data on the control and dystrophic cell lines. Ctl, control; DMD, Duchenne muscular dystrophy; y, year; m, month; del, deletion.

Sample	Pathology	Origin	Age	Sex	Mutation
Ctl1	Control	Paraspinal	12 y	Female	
Ctl2	Control	Paraspinal	13 y	Female	
Ctl3	Control	Quadriceps	53 y	Male	
Ctl4	Control	Quadriceps	38 y	Male	
Ctl5	Control	Paraspinal	16 y	Male	
Ctl6	Control	Triceps	40 y	Male	
DMD1	DMD	Quadriceps	2 y	Male	del 44-50
DMD2	DMD	Biceps	6 y	Male	del 48-50
DMD3	DMD	Quadriceps	20 m	Male	del 48-50
DMD4	DMD	Tensor fascia latae	10 y	Male	del 52
DMD5	DMD	Paravertebral	16 y	Male	del 52

Supplementary Table S2. Composition of used culture media. SGM, Skeletal muscle cell Growth Medium; bDM, basic differentiation medium; cDM, complete differentiation medium; Pro., proliferation; hEGF-DM, human epidermal growth factor differentiation medium.

	**Protocol I**		**Protocol II**	
**Myogenic stage**	Medium	Composition	Medium	Composition
**Proliferation**	SGM	Skeletal muscle cell Growth Medium classic<br>with supplemented mix (PeloBiotech, PB-MH-272-0090)<br>Inactivated 10% fetal bovine serum -FBS- (Gibco™, 10270-106)<br>50 μg/ml gentamicin (Gibco™, 15750037)<br>1X Glutamax (Gibco™, 35050038)	Ultroser Pro.	DMEM High Glucose (Gibco™, 41965039)<br>Inactivated 10% FBS<br>1% Ultroser G serum substitute (Sartorious, 15950-017)<br>MycoZap (Lonza,VZA-2011)
**Differentiation**	bDM	DMEM (Gibco™, 41966029)<br>100 μg/ml apo-Transferrin<br>10 μg/ml insulin (Sigma-Aldrich, I2643-50mg)<br>50 μg/ml gentamicin (Gibco™, 15750037)	hEGF-DM	DMEM High Glucose<br>10 ng/ml human epidermal growth factor<br>(Sigma-Aldrich, E9644)<br>10 μg/ml insulin (Sigma-Aldrich, I9278)<br>50 μg/ml bovine serum albumin (Sigma-Aldrich, A9418)<br>MycoZap
**Maturation**	cDM	Neurobasal A medium (Gibco™, 10888022)<br>B27 1X (Gibco™, 17504044)<br>1X Glutamax<br>20 ng/ml brain-derived neurotrophic factor (Prepotech, 450-02)<br>50 ng/ml sonic hedgehog (Prepotech, 100-45)<br>10 ng/ml insulin-like growth factor 1 (R&D Systems, 4326-RG)<br>5 ng/ml ciliary neurotrophic factor (Prepotech, 450-13)<br>20 ng/ml neurotrophin 3 (Prepotech, 450-03)<br>4 μg/ml laminin (Sigma-Aldrich, L2020)<br>100 ng/ml agrin (R&D Systems, 550-AG-100)<br>50 μg/ml gentamicin	hEGF-DM	
